# Air Jet Impingement–Assisted Air Frying of Potatoes: Oil Absorption, Lipid Oxidation and Acrylamide Formation for Improved Nutritional Quality

**DOI:** 10.1002/fsn3.72017

**Published:** 2026-06-11

**Authors:** Ahmet Tarık Can, Selin Demirkan, Özgül Altay, Özge Filiz, Gönül Çavuşoğlu‐Kaplan, Figen Kaymak‐Ertekin

**Affiliations:** ^1^ Faculty of Engineering, Department of Food Engineering Ege University Izmir Turkey; ^2^ Arçelik A.Ş İstanbul Turkey

**Keywords:** acrylamide, air frying, air jet impingement, lipid oxidation, nutritional quality, oil absorption

## Abstract

Air jet impingement technology has attracted increasing attention in food processing because of its high heat transfer efficiency, which enables rapid heating and improved control of thermally induced chemical reactions. In the present study, frozen French fries and potato wedges were processed using a prototype air jet impingement oven integrated with an air‐frying function and systematically compared with conventional deep‐fat frying and convection oven cooking. The effects of processing temperature, air velocity, tray position, and cooking/frying time on product quality and food safety–related parameters were evaluated using response surface methodology. The investigated quality attributes included moisture content, color development, texture, oil absorption, lipid oxidation (TBARS), sensory acceptance, energy consumption, acrylamide formation, and microstructural characteristics. Compared with deep‐fat frying, air jet impingement–assisted air frying significantly reduced oil content and oil absorption in both French fries and potato wedges while maintaining acceptable textural and sensory properties. The optimum processing conditions were determined as 160°C, 15 m/s, and 15 min for French fries, and 200°C, 15 m/s, and 5 min for potato wedges. Under optimized conditions, acrylamide formation was markedly suppressed, and the acrylamide content of potato wedges remained below the limit of quantification (LOQ = 0.1 mg/kg), as determined by LC–MS/MS analysis. TBARS values and oil absorption increased with increasing air velocity and cooking/frying time; however, optimized impingement conditions minimized lipid oxidation and improved overall product quality. Under optimum air jet impingement conditions, TBARS values were maintained at low levels, reaching 1.812 mg malonaldehyde/kg product for French fries and 2.805 mg malonaldehyde/kg product for potato wedges, corresponding to approximately 43.4% and 16.3% lower lipid oxidation than deep‐fat frying, respectively. In addition, the air jet impingement oven reduced oil absorption by 36.2% in French fries and 38.8% in potato wedges, while decreasing energy consumption by approximately 80% compared with deep‐fat frying. Improved microstructural properties were also observed. Air jet impingement–assisted air frying represents a promising processing strategy for producing potato products with enhanced nutritional quality, reduced formation of harmful thermal contaminants, and improved energy efficiency within a food safety–oriented process control framework.

## Graphical abstract

This study investigates air jet impingement oven cooking as an alternative to conventional frying methods for French fries and potato wedges. The technology improves product quality by enhancing physicochemical and sensory attributes while significantly reducing acrylamide formation. In addition, it offers a faster and more energy‐efficient process compared to deep‐fat frying.

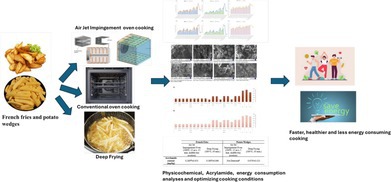

## 1 Introduction

Deep frying is one of the most widely used cooking methods because it rapidly produces foods with highly desirable texture, flavor, and appearance. In this process, food is immersed in edible oils at temperatures typically ranging from 160°C to 180°C. Heat is transferred by convection to the food surface and subsequently conducted to the interior, while simultaneous mass transfer occurs as water diffuses from the center to the surface and evaporates, thereby enabling oil absorption into the product (Alvis et al. 2009; Sahin et al. 1999) Recent studies have demonstrated that modifications in convective heat transfer conditions can significantly influence surface moisture evaporation and frying kinetics, thereby affecting product quality and oil absorption behavior (Li, Zhang, et al. 2025; Li, Zhao, et al. 2025). During frying, numerous physical and chemical transformations occur, including starch gelatinization, protein denaturation, degradation of vitamins and minerals, moisture loss and weight reduction. At the same time, characteristic sensory attributes such as crisp texture, golden‐brown color and desirable aroma compounds are developed (Banerjee and Sahu 2017; Devseren et al. 2016; Hosseini et al. 2016). However, these transformations are also accompanied by the formation of undesirable and potentially harmful compounds such as trans fatty acids, acrylamide, polycyclic aromatic hydrocarbons (PAHs), heterocyclic aromatic amines (HAAs), 3‐monochloropropane‐1,2‐diol (MCPD), and furan (Nerín et al. 2016; Yoon et al. 2024). Excessive consumption of deep‐fried foods has been associated with an increased risk of cancer, coronary heart disease, hypertension, type 2 diabetes, and obesity (Dangal et al. 2024; Verma et al. 2023).

Potatoes are among the most consumed plant‐based foods worldwide after cereals and represent a major source of daily dietary energy intake. They are nutritionally rich in carbohydrates, contain higher protein levels than most root and tuber crops, and are comparable to cereals in terms of protein content (Jaiswal et al. 2024; Karadoğan and Özer 1997). Although potatoes are naturally low in fat and highly versatile, they are typically consumed after cooking to improve digestibility and ensure microbiological safety. Common preparation methods include boiling, baking, microwaving, vacuum‐frying, air‐frying, and deep frying (Çiftçi 2015; Navruz‐Varlı and Mortaş 2023). Frying potatoes, in particular, involves the Maillard reaction between amino acids and reducing sugars, which is responsible for the development of appealing flavor, aroma, and color. Unfortunately, this reaction also contributes to the formation of hazardous thermal contaminants such as acrylamide (AA) and 5‐hydroxymethylfurfural (HMF), both of which are classified as potentially carcinogenic (Ahmed et al. 2023; Çiftçi 2015; Pedreschi 2012; Verma et al. 2023; Zaheer and Akhtar 2016). The kinetics of these reactions are strongly influenced by the heat transfer medium. For instance, forced convection with high‐temperature air can accelerate surface moisture removal and modify microstructural porosity, thereby affecting the formation kinetics of AA and HMF in comparison with conventional deep‐fat frying (Li, Zhang, et al. 2025; Li, Zhao, et al. 2025; Navruz‐Varlı and Mortaş 2023).

In response to these health and safety concerns, alternative cooking technologies have been developed to reduce oil absorption, minimize the formation of harmful compounds, and improve energy efficiency. Among these technologies, air frying has gained considerable attention. Air frying (hot air frying) is based on the rapid circulation of heated air around the food within a closed chamber using a high‐powered fan (Zaghi et al. 2019). Forced convection of high‐temperature air has been reported to accelerate heat and mass transfer, promote rapid moisture evaporation, and contribute to crust formation and structural development in air‐fried products (Li, Zhang, et al. 2025; Li, Zhao, et al. 2025; Yoon et al. 2024). Compared with conventional deep fat frying, air frying requires little or no added oil, produces no waste frying oil, and allows shorter cooking times within a compact processing environment. Despite the reduced oil content, air‐fried foods generally retain favorable nutritional, textural, and browning characteristics (Fabre et al. 2018; Hawa et al. 2024; Liu et al. 2022; Téllez‐Morales et al. 2024). Studies have also shown that hot air frying can improve nutritional quality by minimizing the loss of fat‐soluble vitamins and limiting fat oxidation (Heredia et al. 2014).

Another emerging thermal processing technology with high heat transfer potential is the air jet impingement system. In recent years, jet impingement technology has attracted increasing attention in food processing applications because of its exceptionally high local heat and mass transfer performance (Montevecchi et al. 2018). The system enhances heat transfer by directing high‐velocity air jets (10–100 m/s) onto the food surface through nozzles stabilized by plenums within an enclosed cabinet (Moreira 2001; Sarkar and Singh 2004). Unlike conventional ovens, where a thermal boundary layer limits heat transfer, impingement jets disrupt this layer through intense turbulence and localized recirculation zones, resulting in highly efficient and uniform heating (Altay et al. 2024; Karabulut 2019). As a result, impingement ovens achieve shorter cooking times, improved product texture, enhanced microbial safety, and reduced formation of harmful thermal contaminants (Anderson 2004; Li and Walker 1996). Several studies have confirmed that impingement systems lower energy consumption, limit non‐enzymatic browning, reduce carcinogen formation, and better preserve product color compared to conventional ovens (Patel et al. 2005; Wählby et al. 2000; Yancey et al. 2011).

A promising approach involves the integration of air frying with air jet impingement systems, combining rapid convective heating with intensified surface heat transfer. In this hybrid system, the air‐fry unit provides homogeneous hot air circulation within the processing chamber, while high‐velocity impingement jets directly target the product surface, thereby enhancing heat transfer efficiency and moisture removal. Such an integrated application may offer several advantages, including reduced oil absorption compared with conventional deep fat frying, lower formation of toxic thermal contaminants such as acrylamide and HMF, shorter processing times, improved energy efficiency, and preservation of desirable sensory and textural properties. Although previous studies have separately investigated hot air frying and impingement heating technologies, limited information is available regarding their combined application in fried potato products, particularly with respect to the simultaneous evaluation of oil absorption, lipid oxidation, acrylamide formation, and energy consumption. Furthermore, the interactions between impingement airflow dynamics and air‐fry circulation in relation to thermal contaminant formation and product quality attributes remain insufficiently understood. Therefore, the development and optimization of air jet impingement–assisted air frying systems represent an important research gap in the field of healthier frying technologies.

Accordingly, the present study aimed to evaluate the performance of an air jet impingement oven equipped with an integrated air‐fry function for potato frying applications. The effects of frying temperature, air velocity, and processing time on oil absorption, lipid oxidation, acrylamide formation, physicochemical properties, and energy consumption were systematically investigated. In addition, process optimization was performed to identify frying conditions that minimize oil absorption and the formation of harmful thermal contaminants while maintaining desirable product quality attributes. It was hypothesized that the integration of air jet impingement and air‐fry technology would enhance heat transfer efficiency, reduce processing time and energy consumption, and improve the overall quality and safety of fried potato products.

## 2 Materials and Methods

### 2.1 Materials

Frozen French fries and potato wedges were purchased from a local retail market to reduce variability between batches. The products were stored at −25°C until use in the cooking/frying experiments.

### 2.2 Method

#### 2.2.1 Sample Preparation

Frozen potato samples were removed from cold storage immediately prior to cooking/frying and weighed (300 g) without thawing to ensure a consistent tray load in each experiment. Potato samples were selected to have similar dimensions of 0.9 × 7.6 × 0.9 cm for French fries and 2.5 × 2.0 × 2.5 cm for potato wedges. A total of 9 g of vegetable oil was added to each portion and thoroughly mixed to achieve uniform surface coating. The oil‐coated potatoes were evenly distributed on the air‐fry accessory, and a thermocouple was inserted into the geometric center of one sample for temperature monitoring during cooking/frying.

#### 2.2.2 Cooking/Frying Processes in the Impingement Oven With Air Fry Function

Cooking/frying experiments were carried out using a home‐type impingement oven prototype equipped with an integrated air‐fry accessory, developed by Arçelik A.Ş. The prototype system was designed based on the dimensions and configuration of a commercially available built‐in conventional oven to ensure comparability between the two systems. The oven chamber dimensions were 59.5 × 59.4 × 56.7 cm (height × width × depth). Unlike conventional forced‐convection ovens, the impingement oven was additionally equipped with an air‐jet impingement module consisting of 50 nozzles, including 25 upper and 25 lower nozzles positioned on the top and bottom panels of the oven cavity. Hot air was distributed through these nozzles to provide direct and uniform impingement onto the product surface, thereby enhancing heat and mass transfer during cooking/frying. Flow behavior and airflow distribution analyses of the impingement system were conducted by the manufacturer during the prototype development stage. Based on these analyses, the optimum nozzle diameter was determined to be 13 mm, the nozzle height 6 mm, and the nozzle‐to‐surface distance ratio (H/D) 1:1. The integrated air‐fry accessory was positioned inside the oven cavity, and two tray positions, namely bottom and middle levels, were evaluated. Tray positions were defined according to the vertical distance between the product surface and the lower impingement nozzle array. The nozzle‐to‐product surface distances for the bottom and middle tray positions were measured as 10 and 16 cm, respectively.

For comparison purposes, cooking/frying experiments were also performed using a separate commercially available conventional oven with chamber dimensions and heating capacity comparable to those of the impingement oven system. In contrast to the impingement oven prototype, the conventional oven was not equipped with an air‐jet impingement module and operated solely by means of conventional convective airflow without directional high‐velocity air jets. In addition, deep‐fat frying (180°C for 10 min), conventional oven cooking using a standard baking tray, and conventional oven cooking with an air‐fry accessory (180°C for 10 min) were included as reference treatments for comparative evaluation.

#### 2.2.3 Optimization of Cooking/Frying Processes

Optimization of the cooking/frying process was performed using a Central Composite Rotatable Design (CCRD) within the framework of Response Surface Methodology (RSM). Oven temperature (A), impingement air velocity (B), processing time (C), and tray position (D) were selected as the independent variables. For French fries, processing temperatures of 150°C and 175°C were selected, whereas temperatures of 175°C and 200°C were used for potato wedges. Air velocities of 9, 12, and 15 m/s and processing times of 5, 10, and 15 min were evaluated for both product types. Two tray positions (bottom and middle levels) were also investigated. Separate experimental designs were established for French fries and potato wedges, resulting in 18 treatment combinations for each product type and a total of 36 experimental runs.

Samples obtained after cooking/frying were analyzed for moisture content, water activity, color parameters (b* and browning index [BI]), crust thickness, texture profile analysis (TPA), compression properties, oil content, TBARS value, and sensory attributes.

The response variables selected for optimization were hardness, b* value, TBARS value, and crust thickness (for potato wedges only). Hardness and b* value were selected as indicators of texture and visual quality, whereas TBARS value was used as an indicator of lipid oxidation and oxidative stability. Crust thickness was included only for potato wedges because noticeable differences in crust formation were observed among processing conditions for this product type. In contrast, French fries did not exhibit significant variations in crust thickness under the tested conditions; therefore, crust thickness was excluded from the optimization model for French fries. Other measured parameters, including moisture content, water activity, oil content, texture profile parameters, compression properties, browning index, and sensory attributes, were evaluated for overall product characterization and comparative assessment. However, these parameters were not included in the optimization model to avoid overparameterization and multicollinearity among the response variables.

Experimental design, regression modeling, and optimization analyses were performed using Design‐Expert (Version 13.0.0, Stat‐Ease Inc., USA). Second‐order polynomial regression models were fitted to the experimental data to describe the relationships between process variables and response parameters according to Equation (1):

(1) $$Y=\beta_{0}+\sum \beta_{i}X_{i}+\sum \beta_{\mathrm{ii}}X_{i}^{2}+\sum \beta_{\mathrm{ij}}X_{i}X_{j}$$

where Y is the predicted response, $\beta_{0}$ is the intercept coefficient, $\beta_{i}$ represents the linear coefficients, $\beta_{\mathrm{ii}}$ the quadratic coefficients, and $\beta_{\mathrm{ij}}$ the interaction coefficients. The significance of model terms was evaluated using analysis of variance (ANOVA), while model adequacy was assessed based on the coefficient of determination (*R* ^2^), adjusted *R* ^2^, predicted *R* ^2^, lack‐of‐fit tests, and adequate precision values. Optimization was carried out using the desirability function approach. To validate the optimized processing conditions predicted by the model, three independent production trials were conducted at the optimum point determined by the desirability function analysis. The experimentally obtained response values were compared with the predicted values generated by the regression models to evaluate the adequacy and predictive capability of the developed models.

For comparative evaluation, potato samples processed under the optimized conditions were also compared with reference cooking methods including deep‐fat frying (180°C for 10 min), conventional oven cooking using a standard baking tray, and conventional oven cooking using an air‐fry accessory. To ensure comparable cooking endpoints among treatments, thermocouples were inserted into the geometric center of the potato samples and core temperatures were recorded after cooking/frying. In deep‐fat frying, final core temperatures of 154.1°C and 160.0°C were measured for French fries and potato wedges, respectively. In the conventional oven equipped with the air‐fry accessory, the corresponding core temperatures were 137.2°C for French fries and 142.2°C for potato wedges, whereas values of 137.2°C and 145.2°C were obtained for conventional baking tray cooking.

All cooking/frying experiments were conducted in duplicate, whereas all analytical measurements were performed in triplicate to ensure reproducibility and reliability of the results.

### 2.3 Analysis

#### 2.3.1 Moisture Content

The moisture content of potato samples cooked under different conditions was determined by oven‐drying at 104°C for 4–5 h until a constant weight was achieved. Moisture content (% w/w) was calculated based on weight loss (AOAC 2005).

#### 2.3.2 Cooking Loss

Cooking loss resulting from the cooking/frying process was determined using the gravimetric method. For this purpose, potato samples were weighed before and after the cooking/frying process, and the weight difference was recorded. Cooking loss was expressed as a percentage (%) relative to the initial sample weight (Altay et al. 2024).

#### 2.3.3 Oil Content and Oil Absorption

Oil content (% w/w) was determined using the Soxhlet gravimetric method with hexane as the extraction solvent (Cemeroğlu 2010). Oil absorption (%, dry matter basis) of potatoes was calculated using the formula given in Equation (2) (O'Connor et al. 2001).

(2) $$\begin{array}{c} \text{Oil Absorption}\;\left(\%,\mathrm{DM}\right)=\\ \frac{\left(\text{Oil content after cooking}\right)-\left(\text{Oil content before cooking}\right)}{\text{Oil content before cooking}}\times 100 \end{array}$$

#### 2.3.4 Thiobarbituric Acid Reactive Substances (TBARS) Value

Lipid oxidation products were quantified using the thiobarbituric acid reactive substances (TBARS) assay adapted from Yin et al. (1993). Briefly, 10 g of sample was homogenized with an 11% (w/v) trichloroacetic acid solution and filtered through Whatman No. 1 filter paper. A 5 mL aliquot of the filtrate was mixed with 5 mL of thiobarbituric acid solution and incubated in a water bath at 55°C for 30 min. After cooling, absorbance was measured at 532 nm, and results were expressed as mg malonaldehyde per kg of product.

#### 2.3.5 Crust Thickness

Crust thickness was measured on cooked samples using a digital caliper, following the method described by Pinthus et al. (1995).

#### 2.3.6 Color Characteristics

Color parameters (L*, a*, b*) were measured on the surface of fried samples using a colorimeter (Konica Minolta CR‐300, Japan). The browning index (BI) was calculated according to Equation (3) (Shahabi et al. 2014). Each color measurement was performed in triplicate.

(3) $$\mathrm{BI}=\frac{\left[100\times \left(\frac{a+1.75L}{5.645L+a-3.012b}-0.31\right)\right]}{0.17}$$

#### 2.3.7 Texture Properties

Texture properties were evaluated using a texture analyzer (TAXT2, Stable Micro Systems, Haslemere, UK). Texture profile analysis (TPA) was conducted using a 36R cylindrical probe to determine hardness, while cutting hardness was measured using a Warner–Bratzler blade attachment. All texture analyses were performed in duplicate. Cutting hardness was defined as the maximum force (N) required to fracture and cut through the sample structure, reflecting crust rigidity and internal structural resistance (Bourne 2002; Chang et al. 2011).

#### 2.3.8 Acrylamide Analysis

Acrylamide levels in cooked/fried potato samples were determined by Radix İzmir Private Food Control Laboratory using liquid chromatography–tandem mass spectrometry (LC–MS/MS). Analyses were performed using an in‐house validated analytical method (KAL‐SCY‐217). The limit of quantification (LOQ) of the method was 0.1 mg/kg. Acrylamide concentrations reported below the quantification limit indicate that the acrylamide content of the sample was lower than 0.1 mg/kg.

#### 2.3.9 Scanning Electron Microscopy (SEM)

The morphological characteristics of the samples were examined using a scanning electron microscope (SEM) (FEI Quanta 250 FEG). Samples were mounted on aluminum stubs using double‐sided adhesive tape and sputter‐coated with a thin layer of gold prior to analysis. Coated samples prepared under optimum processing conditions were observed at an accelerating voltage of 5 kV. Images representing both the internal and external microstructures of the samples were obtained and evaluated in conjunction with the SEM analysis.

#### 2.3.10 Sensory Evaluation

Sensory evaluation was conducted using a semi‐trained panel consisting of 10 members selected from undergraduate and graduate students in the field of Food Engineering. Evaluation forms were prepared based on literature data and included the attributes of appearance, color, texture, crust crispness, internal texture, oiliness, and overall acceptability, which were assessed using a nine‐point hedonic scale, where 1 represented “dislike extremely” and 9 represented “like extremely” (Altuğ 2005). Prior to the evaluation, panelists were informed that the samples consisted of frozen pre‐processed French fries and potato wedges, that the target surface color was golden yellow, and that only 3% (w/w) additional oil had been applied apart from the inherent oil content of the products. Panelists were asked to consider these criteria during the sensory evaluation.

Samples were served in randomized order and identified using three‐digit random codes to minimize bias. All samples were served warm immediately after frying under identical conditions. Each panel session included a limited number of samples to prevent sensory fatigue. Panelists were instructed to cleanse their palate with water and unsalted crackers between sample evaluations. Sensory data were analyzed using analysis of variance (ANOVA), followed by Duncan's multiple range test at a significance level of *p* < 0.05.

#### 2.3.11 Energy Consumption

Energy consumption during cooking/frying was measured using an energy analyzer connected between the oven and the power supply line in accordance with International Electrotechnical Commission IEC 60350 standards. Energy usage was recorded throughout each cooking/frying process and expressed as the total electrical energy consumed per cooking batch (kWh). For all cooking methods, the same sample quantity was used; therefore, energy comparisons were performed on a per‐batch basis under standardized processing conditions. The measured energy values represented the total energy consumed during the entire processing period for each treatment condition.

#### 2.3.12 Statistical Analysis

Data was analyzed using analysis of variance (ANOVA) with SPSS software (version 22.0, IBM Corp., Armonk, NY, USA), while process optimization was performed using Design‐Expert software (version 13.0.0, Stat‐Ease Inc., USA). The effects of frying temperature, air velocity, processing time, and tray position on the response variables were evaluated, and optimum cooking/frying conditions were determined using the desirability function approach. Model significance was assessed based on *p* values (*p* < 0.05), and model adequacy was evaluated using determination coefficients (*R* ^2^) and lack‐of‐fit tests. Mean comparisons were performed using Duncan's multiple comparison test at a significance level of *p* < 0.05.

## 3 Results and Discussion

### 3.1 Physical Properties

**FIGURE 1:**
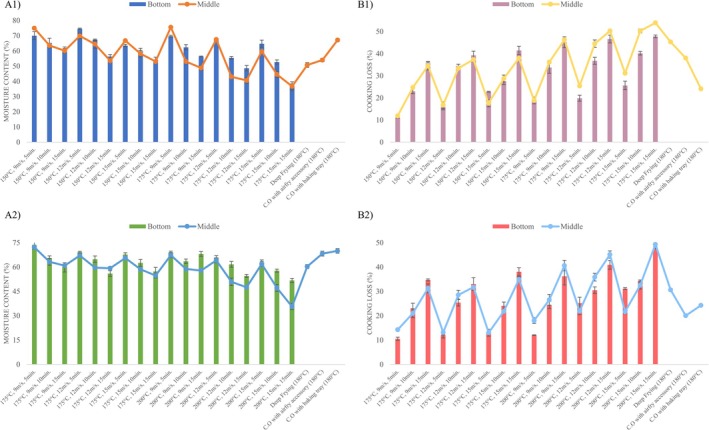
Moisture content and cooking loss of (A1, B1) French Fries, (A2, B2) potato wedges, and control samples. C.O, Conventional Oven.

The moisture content and cooking loss of French fries and potato wedges under different processing conditions are presented in Figure 1, whereas crust thickness, *b* values, and browning index (BI) are summarized in Table 1. The corresponding ANOVA results and regression coefficients are provided in Tables 2 and 3, respectively. As illustrated in Figure 1 and supported by the ANOVA results (Table 2), variations in moisture content were observed between tray positions under different processing conditions. These variations may be associated with differences in local heat and mass transfer conditions within the oven system as well as airflow distribution generated by the impingement nozzle configuration. However, because detailed experimental airflow mapping within the oven cavity was not performed in the present study, this interpretation should be considered with caution. The regression coefficients presented in Table 3 indicated negative relationships between moisture content and oven temperature, air velocity, and processing time. In addition, the perforated structure of the air‐fry accessory may have influenced airflow distribution within the oven cavity. Crust formation, particularly under prolonged cooking conditions, may also affect heat and mass transfer behavior during processing. Processing conditions associated with increased crust formation may have altered internal moisture migration during cooking. Crust thickness is considered an important structural parameter that may influence texture‐related quality characteristics in fried potato products. Previous studies have suggested that crust development can affect crispness perception and mechanical texture properties in fried potato products. However, no direct sensory crispness evaluation or instrumental mechanical texture analysis specifically related to crust structure was performed in the present study.

**TABLE 1:** Crust thickness, *b* value, and browning index (BI) of French fries, potato wedges, and control samples cooked under different processing conditions.

French fries								
Temperature (°C)	Air velocity (m/s)	Time (minute)	Crust thickness (mm)		b*		BI	
			Tray position					
			Bottom	Middle	Bottom	Middle	Bottom	Middle
150	9	5	0.275 ± 0.096	0.350 ± 0.058	17.72 ± 1.83	15.60 ± 0.63	29.67 ± 3.41	27.20 ± 2.71
		10	0.425 ± 0.096	0.425 ± 0.050	21.42 ± 2.91	25.84 ± 1.93	30.01 ± 4.97	40.51 ± 1.68
		15	0.500 ± 0.082	0.450 ± 0.058	23.97 ± 2.84	27.20 ± 4.26	36.55 ± 5.24	44.43 ± 9.88
	12	5	0.325 ± 0.096	0.400 ± 0.082	18.25 ± 1.83	17.80 ± 2.61	30.73 ± 2.66	28.55 ± 5.60
		10	0.475 ± 0.050	0.425 ± 0.096	20.75 ± 0.65	20.71 ± 1.31	31.31 ± 0.95	32.48 ± 3.94
		15	0.600 ± 0.082	0.525 ± 0.050	28.19 ± 2.15	29.34 ± 1.96	47.01 ± 5.32	52.08 ± 6.30
	15	5	0.400 ± 0.082	0.450 ± 0.058	24.89 ± 2.58	24.14 ± 1.69	39.71 ± 4.07	40.83 ± 3.46
		10	0.500 ± 0.082	0.500 ± 0.082	25.96 ± 1.95	25.54 ± 1.33	41.18 ± 4.90	41.61 ± 4.13
		15	0.525 ± 0.096	0.575 ± 0.096	26.20 ± 2.14	27.33 ± 0.68	44.17 ± 3.44	45.62 ± 2.61
175	9	5	0.375 ± 0.096	0.400 ± 0.082	23.20 ± 1.47	21.24 ± 4.84	35.84 ± 5.36	32.95 ± 7.08
		10	0.400 ± 0.001	0.450 ± 0.058	21.61 ± 2.10	22.33 ± 3.24	39.01 ± 4.86	40.15 ± 8.93
		15	0.650 ± 0.058	0.500 ± 0.082	28.55 ± 0.69	28.69 ± 5.24	53.30 ± 5.62	55.05 ± 15.05
	12	5	0.425 ± 0.096	0.425 ± 0.096	22.12 ± 1.62	22.70 ± 4.66	35.58 ± 2.54	35.51 ± 8.62
		10	0.525 ± 0.096	0.475 ± 0.096	31.98 ± 1.56	33.73 ± 3.56	51.58 ± 1.66	62.70 ± 7.73
		15	0.725 ± 0.096	0.525 ± 0.050	28.82 ± 1.72	27.47 ± 2.04	54.22 ± 6.83	50.81 ± 0.91
	15	5	0.525 ± 0.050	0.550 ± 0.058	24.83 ± 1.30	24.68 ± 1.84	38.09 ± 4.00	40.70 ± 3.98
		10	0.625 ± 0.096	0.600 ± 0.082	24.64 ± 0.36	24.19 ± 0.96	44.12 ± 3.15	42.88 ± 5.28
		15	0.800 ± 0.082	0.875 ± 0.050	30.27 ± 1.60	34.75 ± 0.86	53.47 ± 4.43	71.70 ± 3.12
Deep Fried (180°C)		10		0.588 ± 0.136		34.53 ± 1.93		71.02 ± 2.71
C.O with air fry accessory (180°C)				0.450 ± 0.129		29.29 ± 2.28		48.37 ± 3.13
C.O with baking tray (180°C)				0.275 ± 0.050		28.26 ± 1.32		47.98 ± 2.28

Potato wedges								
Temperature (°C)	Air velocity (m/s)	Time (minute)	Crust thickness (mm)		b*		BI	
			Tray position					
			Bottom	Middle	Bottom	Middle	Bottom	Middle
175	9	5	0.225 ± 0.050	0.250 ± 0.058	20.40 ± 1.37	22.22 ± 2.20	27.32 ± 1.76	30.42 ± 3.61
		10	0.375 ± 0.096	0.450 ± 0.058	24.30 ± 1.27	26.37 ± 2.02	34.38 ± 2.63	42.47 ± 3.46
		15	0.475 ± 0.050	0.525 ± 0.050	25.63 ± 1.42	26.87 ± 1.26	41.38 ± 2.17	47.45 ± 1.62
	12	5	0.242 ± 0.012	0.271 ± 0.006	21.76 ± 0.86	23.08 ± 1.69	33.12 ± 1.51	33.95 ± 3.12
		10	0.425 ± 0.050	0.475 ± 0.050	22.66 ± 0.61	27.47 ± 1.55	39.12 ± 1.56	46.58 ± 2.38
		15	0.479 ± 0.065	0.546 ± 0.029	26.63 ± 0.94	29.23 ± 0.20	42.72 ± 1.77	49.82 ± 1.75
	15	5	0.271 ± 0.006	0.308 ± 0.059	20.21 ± 0.72	22.29 ± 0.77	34.36 ± 1.17	34.47 ± 1.91
		10	0.454 ± 0.029	0.471 ± 0.006	24.80 ± 0.88	25.46 ± 1.69	41.05 ± 3.76	39.99 ± 2.26
		15	0.496 ± 0.041	0.567 ± 0.047	29.51 ± 0.97	29.60 ± 0.91	62.74 ± 1.91	61.08 ± 1.62
200	9	5	0.300 ± 0.071	0.263 ± 0.018	21.00 ± 0.44	22.68 ± 2.05	32.17 ± 1.12	39.16 ± 3.97
		10	0.383 ± 0.024	0.467 ± 0.047	23.24 ± 1.41	25.96 ± 1.42	35.95 ± 2.84	44.69 ± 3.61
		15	0.579 ± 0.077	0.633 ± 0047	25.49 ± 0.49	26.87 ± 0.39	44.75 ± 3.35	50.57 ± 3.07
	12	5	0.333 ± 0.047	0.304 ± 0.041	23.16 ± 1.95	25.37 ± 0.56	34.33 ± 4.58	41.52 ± 1.19
		10	0.433 ± 0.000	0.500 ± 0.082	26.02 ± 0.26	29.97 ± 0.94	42.65 ± 1.47	53.87 ± 1.55
		15	0.600 ± 0.047	0.717 ± 0.118	31.58 ± 1.43	30.08 ± 2.36	55.41 ± 3.85	54.60 ± 3.90
	15	5	0.342 ± 0.153	0.333 ± 0.094	25.36 ± 1.84	27.26 ± 0.99	43.38 ± 3.88	43.11 ± 2.40
		10	0.458 ± 0.012	0.588 ± 0.018	26.75 ± 0.79	31.67 ± 1.21	48.98 ± 1.07	54.55 ± 2.57
		15	0.613 ± 0.124	0.767 ± 0.058	33.66 ± 1.56	33.20 ± 1.68	65.51 ± 2.40	77.18 ± 1.29
Deep Fried (180°C)		10		0.550 ± 0.141		28.42 ± 1.22		61.02 ± 2.93
C.O with air fry accessory (180°C)				0.275 ± 0.050		23.87 ± 0.50		45.40 ± 1.07
C.O with baking tray (180°C)				0.240 ± 0.055		19.28 ± 1.04		30.94 ± 2.25

Abbreviation: C.O., conventional oven.

**TABLE 2:** Analysis of variance (ANOVA) for moisture content, cooking loss, crust thickness, *b* value, and browning index (BI) of French Fries and potato wedges cooked under different processing conditions.

French fries											
Source	DF	Moisture content (%)		Cooking loss (%)		Crust thickness (mm)		*b*		BI	
		Sum of squares	*p*	Sum of squares	*p*	Sum of squares	*p*	Sum of squares	*p*	Sum of squares	*p*
Modal	12	3485.61	< 0.0001	4692.44	< 0.0001	0.4907	< 0.0001	462.37	0.0017	2711.81	< 0.0001
A	1	505.91	< 0.0001	299.91	< 0.0001	0.1240	< 0.0001	66.83	0.0136	262.75	0.0114
B	1	654.00	< 0.0001	877.44	< 0.0001	0.0827	< 0.0001	83.88	0.0065	658.86	0.0002
C	1	1981.80	< 0.0001	3272.04	< 0.0001	0.2301	< 0.0001	291.28	< 0.0001	1552.85	< 0.0001
D	1	72.19	0.0209	32.09	0.0388	0.0009	0.5608	2.73	0.5942	70.03	0.1693
AB	1	41.53	0.0727	8.50	0.2711	0.0190	0.0105	0.8702	0.7630	4.24	0.7301
AC	1	6.54	0.4629	17.33	0.1209	0.0014	0.4557	7.08	0.3933	4.04	0.7363
AD	1	0.6112	0.8215	4.02	0.4459	0.0021	0.3624	0.0145	0.9689	1.86	0.8189
BC	1	124.90	0.0034	16.02	0.1351	0.0067	0.1121	0.6834	0.7893	90.91	0.1196
BD	1	36.76	0.0900	69.47	0.0038	0.0029	0.2844	0.1587	0.8975	0.5065	0.9050
CD	1	26.31	0.1479	0.3577	0.8191	0.0150	0.0210	7.74	0.3723	50.61	0.2400
A^2^	1	0.3712	0.8604	17.70	0.1172	0.0025	0.3214	1.11	0.7338	3.72	0.7465
C^2^	1	34.68	0.0990	77.56	0.0024	0.0035	0.2453	0.0042	0.9833	11.43	0.5720
Residual	23	269.91		153.66		0.0562		215.00		799.87	
Cor total	35	3755.51		4846.10		0.5469		677.36		3511.68	

Potato wedges											
Source	DF	Moisture content (%)		Cooking loss (%)		Crust thickness (mm)		b*		BI	
		Sum of squares	*p*	Sum of squares	*p*	Sum of squares	*p*	Sum of squares	*p*	Sum of squares	*p*
Modal	12	1849.62	< 0.0001	3756.02	< 0.0001	0.6504	< 0.0001	409.95	< 0.0001	318.07	< 0.0001
A	1	307.59	< 0.0001	198.66	< 0.0001	0.0230	< 0.0001	62.53	< 0.0001	767.16	< 0.0001
B	1	285.67	< 0.0001	630.43	< 0.0001	0.0475	< 0.0001	46.31	< 0.0001	399.73	< 0.0001
C	1	970.41	< 0.0001	2774.58	< 0.0001	0.5266	< 0.0001	225.46	< 0.0001	2126.28	< 0.0001
D	1	131.22	0.0001	0.6917	0.7648	0.0252	< 0.0001	31.16	< 0.0001	206.21	0.0006
AB	1	34.99	0.0265	100.08	0.0014	0.0018	0.1657	29.44	< 0.0001	51.48	0.0586
AC	1	11.14	0.1942	0.8281	0.7434	0.0000	0.9010	9.44	0.0087	196.77	0.0007
AD	1	31.51	0.0344	28.10	0.0660	0.0009	0.3180	0.1233	0.7459	24.91	0.1795
BC	1	14.21	0.1445	0.2420	0.8594	0.0110	0.0019	0.0888	0.7832	0.3267	0.8754
BD	1	41.50	0.0167	4.60	0.4428	0.0003	0.5445	0.0003	0.9865	18.89	0.2402
CD	1	6.15	0.3307	0.6501	0.7717	0.0103	0.0024	2.44	0.1577	4.37	0.5677
A^2^	1	2.01	0.5752	14.46	0.1795	0.0000	0.8236	2.43	0.1591	6.56	0.4846
C^2^	1	13.22	0.1587	2.70	0.5559	0.0037	0.0517	0.5339	0.5018	14.19	0.3070
Residual	23	143.24		173.54		0.0204		26.36		13.00	
Cor total	35	1992.86		3929.56		0.6708		436.32			

*Note:* A: Air velocity, B: Temperature, C: Time, D: Location (Tray position), Different letters in the same column are significant at *p* < 0.05.

**TABLE 3:** Regression coefficients of coded factors for moisture content, cooking loss, crust thickness, *b* value, and browning index of French fries and potato wedges.

French fries					
	Coefficient estimate				
	Moisture content (%)	Cooking loss (%)	Crust thickness (mm)	b*	BI
Intercept	57.60	35.45	0.4736	25.14	41.92
A	−4.59	3.53	0.0719	1.67	3.31
B	−4.26	4.94	0.0479	1.53	4.28
C	−9.09	11.68	0.0979	3.48	8.04
D	−1.42	0.9442	−0.0049	0.2753	1.39
AB	−1.32	0.5950	0.0281	−0.1904	−0.4204
AC	−0.6394	−1.04	0.0094	−0.6650	−0.5025
AD	−0.1596	0.4092	0.0094	−0.0246	0.2788
BC	−2.28	0.8171	0.0167	−0.1687	1.95
BD	−1.01	1.39	−0.0090	−0.0664	0.1186
CD	−1.05	−0.1221	−0.0250	0.5679	1.45
A^2^	−0.2154	−1.49	0.0177	−0.3721	−0.6821
C^2^	2.08	−3.11	0.0208	0.0229	1.20

Potato wedges					
	Coefficient estimate				
	Moisture content (%)	Cooking loss (%)	Crust thickness (mm)	b*	BI
Intercept	59.17	28.23	0.4582	26.59	43.09
A	−3.58	2.88	0.0310	1.61	5.65
B	−2.82	4.18	0.0363	1.13	3.33
C	−6.36	10.75	0.1481	3.06	9.41
D	−1.91	0.1386	0.0264	0.9303	2.39
AB	−1.21	2.04	0.0087	1.11	1.46
AC	−0.8344	0.2275	0.0009	0.7681	3.51
AD	−1.15	−1.08	0.0062	−0.0717	−1.02
BC	−0.7696	0.1004	0.0214	−0.0608	0.1167
BD	−1.07	0.3575	0.0031	0.0031	0.7244
CD	−0.5062	0.1646	0.0207	−0.3192	0.4267
A^2^	0.5017	−1.34	−0.0024	−0.5508	0.9054
C^2^	1.29	0.5804	−0.0216	−0.2583	1.33

*Note:* A: Air velocity, B: Temperature, C: Time, D: Location (Tray position).

Compared with the control treatments, noticeable differences in moisture retention and cooking loss were observed in samples processed using the air jet impingement oven, with some variations detected between tray positions. The high heat transfer efficiency of the impingement system, particularly when combined with extended cooking times, accelerated surface drying and promoted crust formation. Consequently, crust thickness varies depending on the processing conditions and cooking method applied. Increased crust formation may have influenced moisture migration behavior during processing, thereby affecting both cooking loss and residual moisture content. Similar observations were reported by Andrés et al. (2013), who compared untreated, frozen, and boiled potato samples processed by air frying, deep‐fat frying, and conventional cooking methods.

Color is a critical sensory attribute for fried potato products, as the development of a golden‐yellow appearance is highly valued by consumers. This characteristic color is primarily determined by Maillard reactions between reducing sugars and amino acids, which are influenced by drying rate, heat transfer efficiency, product composition, frying temperature, and processing time. The *b* value, which represents yellow color intensity, is a key indicator of color quality in fried potato products. According to Heredia et al. (2014), optimal consumer acceptance of fried potatoes is generally associated with *b* values greater than 10. In the present study, potato samples processed in the impingement air jet oven exhibited favorable b* values, although differences were observed between impingement‐cooked and deep‐fat fried samples. Comparable b* ranges were obtained under certain impingement cooking conditions; however, longer processing times were required to achieve similar values at the bottom tray position. As presented in Table 1, browning index (BI) values differed between impingement‐cooked and deep‐fat fried samples. For potato wedges, the regression coefficients indicated positive relationships between BI values and processing intensity variables, particularly processing time. Under the most intensive processing conditions (200°C, 15 m/s air velocity, and 15 min), BI values reached levels comparable to or higher than those observed in deep‐fat fried samples at both tray positions. In contrast, moderate processing conditions (175°C, 15 m/s, and 15 min) produced BI values like those obtained by deep‐fat frying.

In conventional oven trials, both the air fry accessory and conventional baking trays were evaluated. Across all processing conditions, differences in *b* values were observed between impingement and conventional oven treatments. Similarly BI values obtained from conventional oven cooking were generally lower than those observed for deep frying but remained below the levels achieved with impingement air jet cooking under higher‐intensity conditions.

Examination of the statistical analysis results (Table 2) indicated that the investigated response variables (moisture content, cooking loss, crust thickness, b, and BI values) were significantly affected by the tested independent factors (*p* < 0.05). In both potato wedges and French fries, most response variables were significantly influenced by air velocity, temperature, and cooking/frying time (*p* < 0.05). Regression coefficients presented in Table 3 further provided information regarding the direction and relative magnitude of the effects of the processing variables on the investigated response parameters. However, the effect of tray position was not significant for some response variables (*p* > 0.05). Specifically, tray position did not significantly affect crust thickness, *b*, or BI values in French fries, nor cooking loss in potato wedges.

To evaluate the overall performance of the developed models, the coefficients of determination (*R* ^2^) and adjusted coefficients of determination (*R* ^2^_adj_) were examined. The results indicated that most models exhibited a strong fit to the experimental data. For French Fries, strong model adequacy was obtained for moisture content, cooking loss, and crust thickness (*R* ^2^_adj_ = 0.84–0.95), whereas lower *R* ^2^_adj_ values were observed for b and BI. In contrast, all models developed for potato wedges exhibited strong explanatory power, with *R* ^2^_adj_ values ranging from 0.89 to 0.95. Overall, the adjusted *R* ^2^ values confirmed strong model adequacy for potato wedges and for moisture‐related responses in French Fries. In contrast, the models for b and BI in French Fries explained a smaller proportion of the variability, indicating more limited model adequacy for color‐related responses.

### 3.2 Chemical Properties

**FIGURE 2:**
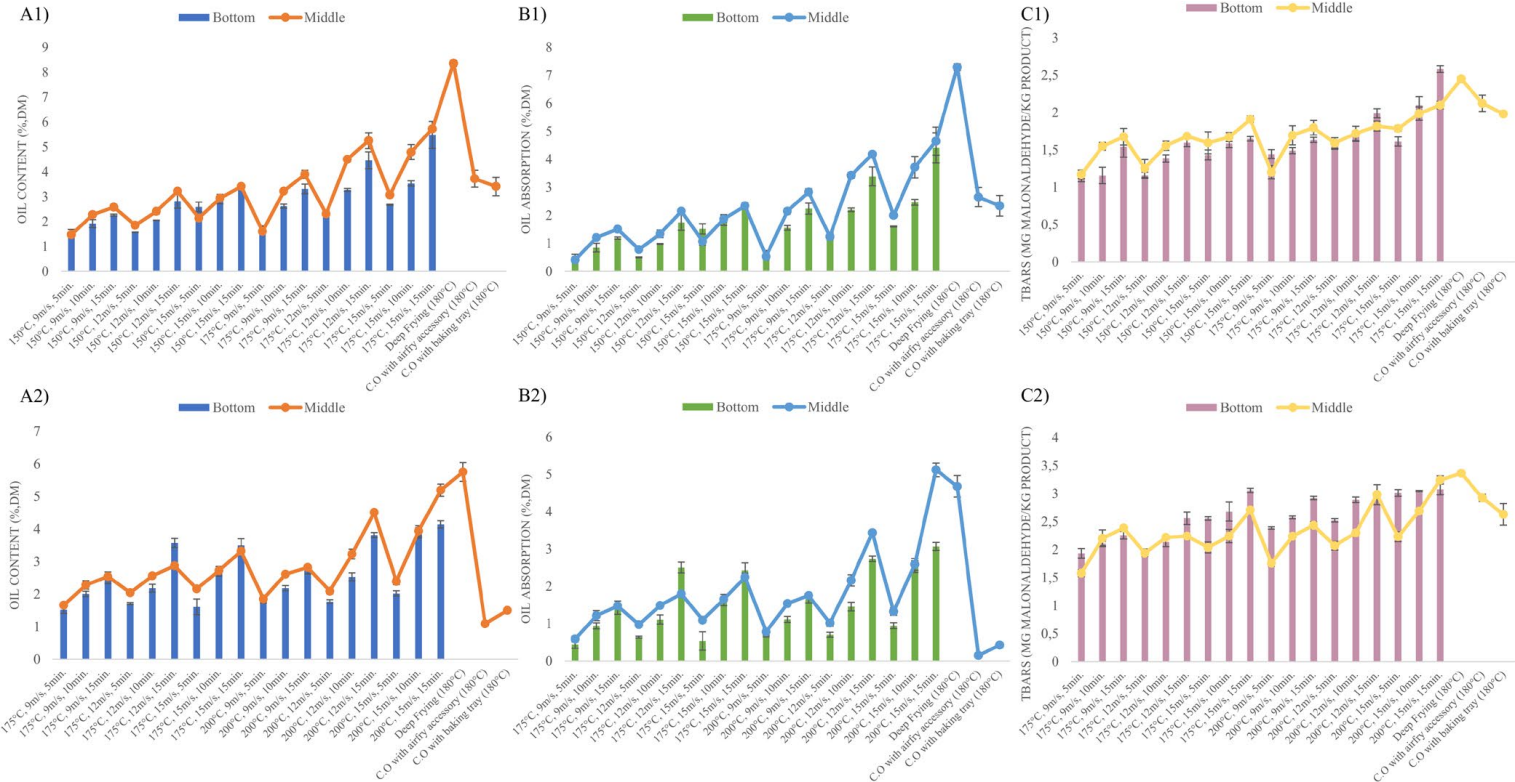
Oil content, oil absorption and TBARS values of (A1, B1, C1) French Fries, (A2, B2, C2) potato wedges, and control samples. C.O, Conventional Oven.

The oil content (g/100 g dry matter), oil absorption (% dry matter), and TBARS values (mg malondialdehyde/kg product) of potatoes processed in the air jet impingement oven with the air‐fry function, together with the control groups, are presented in Figure 2. The ANOVA results illustrating the individual, quadratic, and interaction effects of processing variables on oil content, oil absorption, and TBARS values of French Fries and potato wedges are summarized in Table 4, while the estimated regression coefficients of the developed quadratic models are presented in Table 5.

**TABLE 4:** Analysis of variance (ANOVA) for oil content, TBARS, and oil absorption values of French Fries and potato wedges cooked under different processing conditions.

French fries							
Source	DF	Oil content (%, DM)		Oil absorption (%, DM)		TBARS (mg malonaldehyde/kg product)	
		Sum of squares	*p*	Sum of squares	*p*	Sum of squares	*p*
Modal	12	42.93	< 0.0001	42.97	< 0.0001	2.89	< 0.0001
A	1	8.28	< 0.0001	8.30	< 0.0001	0.8577	< 0.0001
B	1	11.55	< 0.0001	11.57	< 0.0001	0.7253	< 0.0001
C	1	18.06	< 0.0001	18.07	< 0.0001	1.06	< 0.0001
D	1	1.02	0.0014	1.02	0.0014	0.0305	0.1556
AB	1	0.5163	0.0166	0.5163	0.0164	0.0673	0.0397
AC	1	0.2500	0.0854	0.2495	0.0851	0.0005	0.8468
AD	1	0.0003	0.9537	0.0003	0.9478	0.0163	0.2947
BC	1	2.56	< 0.0001	2.56	< 0.0001	0.0061	0.5166
BD	1	0.3823	0.0363	0.3790	0.0368	0.0949	0.0164
CD	1	0.2481	0.0865	0.2468	0.0867	0.0032	0.6379
A^2^	1	0.0103	0.7189	0.0103	0.7178	0.0306	0.1549
C^2^	1	0.0556	0.4055	0.0573	0.3973	0.0008	0.8188
Residual	23	1.78		1.77		0.3256	
Cor total	35	44.71		44.75		3.22	

Potato wedges							
Source	DF	Oil content (%, DM)		Oil absorption (%, DM)		TBARS (mg malonaldehyde/kg product)	
		Sum of squares	*p*	Sum of squares	*p*	Sum of squares	*p*
Modal	12	26.15	< 0.0001	29.98	< 0.0001	5.56	< 0.0001
A	1	5.14	< 0.0001	5.54	< 0.0001	1.40	< 0.0001
B	1	2.78	< 0.0001	3.02	< 0.0001	1.17	< 0.0001
C	1	14.88	< 0.0001	16.49	< 0.0001	1.93	< 0.0001
D	1	0.6032	0.0095	0.8952	0.0103	0.7448	< 0.0001
AB	1	0.7038	0.0056	0.8600	0.0117	0.0012	0.8227
AC	1	1.10	0.0009	1.67	0.0009	0.0006	0.8781
AD	1	0.0273	0.5529	0.1379	0.2839	0.0203	0.3616
BC	1	0.5704	0.0114	0.9200	0.0094	0.0097	0.5267
BD	1	0.2434	0.0855	0.4297	0.0651	0.0808	0.0761
CD	1	0.0253	0.5676	0.0018	0.9027	0.1614	0.0152
A^2^	1	0.0406	0.4704	0.0219	0.6656	0.0411	0.1984
C^2^	1	0.0338	0.5098	0.0040	0.8533	0.0006	0.8706
Residual	23	1.73		2.63		0.5389	
Cor total	35	27.88		32.61		6.10	

*Note:* A: Air velocity, B: Temperature, C: Time, D: Location (Tray position), Different letters in the same column are significant at *p* < 0.05.

**TABLE 5:** Regression coefficients of coded factors for oil content, oil absorption, and TBARS values of French fries and potato wedges.

French Fries			
	Coefficient estimate		
	Oil content (%, DM)	Oil absorption (%, DM)	TBARS (mg malonaldehyde/kg product)
Intercept	3.06	1.99	1.59
A	0.5875	0.5879	0.1890
B	0.5664	0.5668	0.1419
C	0.8675	0.8678	0.2102
D	0.1681	0.1680	0.0291
AB	0.1467	0.1467	0.0530
AC	0.1250	0.1249	0.0058
AD	−0.0033	−0.0037	−0.0260
BC	0.3267	0.3269	0.0160
BD	0.1031	0.1026	−0.0513
CD	0.1017	0.1014	−0.0116
A^2^	−0.0358	−0.0359	0.0619
C^2^	−0.0833	−0.0847	−0.0097

Potato wedges			
	Coefficient estimate		
	Oil content (%, DM)	Oil absorption (%, DM)	TBARS (mg malonaldehyde/kg product)
Intercept	2.78	1.65	2.39
A	0.4629	0.4804	0.2416
B	0.2778	0.2896	0.1802
C	0.7875	0.8288	0.2835
D	0.1294	0.1577	−0.1438
AB	0.1712	0.1893	0.0071
AC	0.2619	0.3227	−0.0059
AD	0.0338	0.0758	−0.0291
BC	0.1542	0.1958	0.0201
BD	0.0822	0.1092	−0.0474
CD	−0.0325	0.0085	0.0820
A^2^	−0.0712	−0.0524	0.0717
C^2^	−0.0650	0.0224	0.0089

*Note:* A: Air velocity, B: Temperature, C: Time, D: Location (Tray position).

As shown in Figure 2, the oil content of potato wedges was 5.75 g/100 g DM, whereas that of French Fries reached 8.36 g/100 g DM under deep frying conditions. In contrast, the oil content of samples processed in the air jet impingement oven with the air‐fry function was significantly lower than that of deep‐fried samples. Similar findings were reported by Teruel et al. (2015), who attributed the reduced oil content of air‐fried products to differences in frying environments. Likewise, Andrés et al. (2013) reported that although air frying required longer cooking/frying times than deep frying, oil absorption was substantially lower. This reduction is attributed to the minimal oil exposure in oven‐based cooking, whereas in deep frying, oil serves as the primary heat transfer medium. Additionally, previous studies (Tarmizi and Niranjan 2010; Ufheil and Escher 1996) have shown that a significant portion of total oil absorption during deep frying occurs after frying, as cooling induces suction‐driven pressure gradients between the product surface and core once temperatures fall below the boiling point of water. Consistent with these results, the oil absorption values determined for both potato wedges and French Fries were significantly reduced when processed in the air jet impingement oven with the air‐fry function compared with deep frying, confirming the lower oil absorption capacity of air‐fried products (Figure 2). Unlike deep frying, where oil acts as the primary heat transfer medium, air jet impingement cooking limits direct oil–food contact, thereby reducing capillary‐driven oil absorption during cooling.

Figure 2 also demonstrates that oil content increased with increasing oven temperature, air velocity, and cooking/frying time. Regression coefficient analysis (Table 5) indicated that cooking/frying time exerted the strongest positive effect on oil content and oil absorption in both French Fries and potato wedges, followed by temperature and air velocity. Similarly, TBARS values, which indicate secondary lipid oxidation, increased significantly with increasing temperature and cooking duration. Positive regression coefficients obtained for temperature, air velocity, and cooking/frying time confirmed that increasing processing intensity promoted lipid oxidation and TBARS formation (Table 5). Lipid oxidation, a common degradation reaction in edible oils, accelerates at elevated cooking temperatures, resulting in the formation of off flavors such as bitterness. The TBARS assay used in this study confirmed that higher temperatures and prolonged cooking/frying conditions enhanced oxidative degradation, consistent with earlier observations (Selçuk et al. 2026).

For French Fries, the oil content of samples processed in the air jet impingement oven with the air‐fry function was lower at both the middle and bottom tray positions compared with those processed in a conventional oven. TBARS values, however, were comparable between the two methods. Overall, these findings confirm that the air jet impingement oven with the air‐fry function effectively reduced both oil content and TBARS values compared with deep frying, aligning with previous studies emphasizing the benefits of this technology in minimizing lipid oxidation. The lower TBARS values observed under impingement‐assisted air frying indicate suppressed secondary lipid oxidation, likely due to reduced oil availability and shorter exposure to high‐temperature oxidative environments, which is relevant for limiting oxidation‐related quality deterioration and improving product stability.

Statistical analysis (Table 3) indicated that oil content, oil absorption, and TBARS values were significantly affected by temperature, air velocity, and cooking/frying time in both potato wedges and French Fries (*p* < 0.05). In addition, the regression coefficient estimates presented in Table 5 provided information regarding the direction and relative magnitude of the effects of each processing parameter and their interactions on the chemical properties investigated. Tray position significantly influenced oil content and oil absorption, whereas its effect on TBARS values was not significant for French Fries (*p* > 0.05). The developed models exhibited strong predictive performance, with adjusted *R* ^2^ values ranging from 0.84 to 0.93, confirming their suitability for describing chemical quality changes during impingement‐assisted air frying.

### 3.3 Textural, Sensory Properties and Energy Consumption

The textural properties (hardness and cutting hardness), together with sensory attributes including color, appearance, crispiness, internal texture, oiliness, and overall sensory acceptability of French fries and potato wedges processed in the air jet impingement oven and control treatments, are presented in Table 6, whereas the corresponding ANOVA and regression analyses are summarized in Tables 7 and 8, respectively.

**TABLE 6:** Hardness, cutting hardness, and sensory attributes (color, appearance, crispiness, internal texture, oiliness and general appreciation) of French fries, potato wedges, and control samples cooked under different processing conditions.

French fries																		
Temperature (°C)	Air velocity (m/s)	Time (minute)	Hardness (N)		Cutting hardness (N)		Color		Appearance		Crispiness		Internal texture		Oiliness		General sensory appreciation	
			Tray position															
			Bottom	Middle	Bottom	Middle	Bottom	Middle	Bottom	Middle	Bottom	Middle	Bottom	Middle	Bottom	Middle	Bottom	Middle
150	9	5	27.48 ± 0.57	31.36 ± 0.86	9.51 ± 0.06	9.65 ± 0.17	6.25 ± 0.50	6.00 ± 0.82	5.50 ± 0.58	5.75 ± 0.50	1.25 ± 0.50	1.50 ± 0.58	1.50 ± 0.58	1.75 ± 0.50	4.75 ± 0.50	4.75 ± 0.50	3.00 ± 0.01	4.75 ± 0.50
		10	30.55 ± 0.55	32.64 ± 0.28	9.60 ± 0.07	9.99 ± 0.70	6.75 ± 0.50	7.00 ± 0.82	6.25 ± 0.50	6.25 ± 0.50	2.50 ± 0.58	2.50 ± 0.58	2.00 ± 0.82	2.00 ± 0.82	2.00 ± 0.82	2.00 ± 0.82	3.25 ± 0.50	5.25 ± 0.50
		15	34.65 ± 0.84	34.13 ± 0.50	10.18 ± 0.50	10.26 ± 0.77	5.00 ± 0.01	6.50 ± 0.58	5.50 ± 0.58	5.75 ± 0.58	4.00 ± 0.01	4.00 ± 0.01	3.25 ± 0.50	3.25 ± 0.50	4.00 ± 0.01	4.00 ± 0.01	3.75 ± 0.50	5.50 ± 0.58
	12	5	29.76 ± 0.70	31.93 ± 0.52	9.63 ± 0.04	9.72 ± 0.03	6.00 ± 0.01	6.25 ± 0.50	4.75 ± 0.50	5.25 ± 0.50	4.00 ± 0.01	4.25 ± 0.50	3.75 ± 0.96	4.00 ± 0.82	1.25 ± 0.50	2.25 ± 0.50	4.50 ± 0.58	5.75 ± 0.50
		10	31.05 ± 0.56	33.27 ± 0.65	10.68 ± 0.23	10.09 ± 0.10	7.00 ± 0.01	7.00 ± 0.01	5.75 ± 0.82	5.75 ± 0.96	5.00 ± 0.01	5.00 ± 0.01	4.75 ± 0.50	4.75 ± 0.50	3.00 ± 0.82	3.00 ± 0.82	5.50 ± 0.58	6.25 ± 0.50
		15	36.60 ± 0.57	34.38 ± 0.67	11.47 ± 0.39	10.33 ± 0.28	8.00 ± 0.01	7.75 ± 0.50	7.00 ± 0.82	7.00 ± 0.82	6.00 ± 0.01	6.00 ± 0.01	5.25 ± 0.96	5.25 ± 0.96	4.25 ± 0.50	4.25 ± 0.50	6.75 ± 0.50	7.50 ± 0.58
	15	5	31.16 ± 0.90	32.28 ± 0.26	10.07 ± 0.18	10.39 ± 0.35	7.00 ± 0.01	6.75 ± 0.50	5.75 ± 0.50	5.75 ± 0.50	5.00 ± 0.01	5.00 ± 0.01	4.75 ± 0.96	4.75 ± 0.96	2.25 ± 0.50	2.25 ± 0.50	7.00 ± 0.01	6.50 ± 0.58
		10	32.05 ± 0.87	33.58 ± 0.51	10.95 ± 0.34	10.59 ± 0.44	8.00 ± 0.01	7.75 ± 0.50	6.75 ± 0.96	6.50 ± 0.58	6.00 ± 0.01	5.75 ± 0.50	5.75 ± 0.50	5.75 ± 0.50	4.00 ± 0.82	4.00 ± 0.82	7.75 ± 0.50	7.75 ± 0.50
		15	37.55 ± 0.82	34.49 ± 0.39	12.11 ± 0.59	10.72 ± 0.10	7.00 ± 0.01	7.25 ± 0.50	5.50 ± 0.58	5.75 ± 0.50	4.50 ± 0.58	5.50 ± 0.58	4.25 ± 0.96	4.25 ± 0.96	3.25 ± 0.50	3.25 ± 0.50	8.50 ± 0.58	9.00 ± 0.01
175	9	5	31.52 ± 0.67	30.76 ± 0.59	9.96 ± 0.14	10.26 ± 0.08	6.25 ± 0.50	6.50 ± 0.58	5.50 ± 0.58	5.75 ± 0.50	1.25 ± 0.50	1.75 ± 0.50	1.50 ± 0.58	2.25 ± 0.50	4.75 ± 0.50	5.00 ± 0.82	5.25 ± 0.50	6.75 ± 0.50
		10	32.28 ± 0.40	32.40 ± 0.27	10.23 ± 0.02	10.63 ± 0.16	6.50 ± 0.58	6.75 ± 0.50	6.75 ± 0.50	6.75 ± 0.50	2.75 ± 0.50	3.00 ± 0.82	2.50 ± 0.58	2.75 ± 0.50	4.75 ± 0.50	5.25 ± 0.50	6.25 ± 0.50	7.00 ± 0.01
		15	34.38 ± 0.39	34.30 ± 0.47	11.37 ± 0.09	10.86 ± 0.21	6.75 ± 0.50	7.00 ± 0.82	6.50 ± 0.58	6.50 ± 0.58	3.75 ± 0.50	4.00 ± 0.82	4.00 ± 0.82	4.00 ± 0.82	5.50 ± 0.58	5.75 ± 0.50	6.75 ± 0.50	8.50 ± 0.58
	12	5	33.98 ± 0.58	32.38 ± 0.65	10.13 ± 0.14	10.86 ± 0.41	5.50 ± 0.58	5.75 ± 0.50	5.75 ± 0.50	5.75 ± 0.50	2.25 ± 0.50	2.50 ± 0.58	2.25 ± 0.50	2.75 ± 0.50	3.75 ± 0.50	4.00 ± 0.82	5.75 ± 0.50	6.50 ± 0.58
		10	34.33 ± 0.49	33.98 ± 0.64	11.12 ± 0.37	11.10 ± 0.16	6.75 ± 0.50	7.00 ± 0.01	6.75 ± 0.50	7.00 ± 0.82	2.75 ± 0.50	3.00 ± 0.82	4.00 ± 0.58	4.50 ± 0.58	5.25 ± 0.50	5.25 ± 0.50	6.75 ± 0.50	7.25 ± 0.50
		15	34.81 ± 0.66	35.83 ± 0.50	11.81 ± 0.13	11.77 ± 0.24	7.50 ± 0.58	7.75 ± 0.50	7.00 ± 0.01	7.25 ± 0.50	5.25 ± 0.50	5.50 ± 0.58	5.50 ± 0.58	5.75 ± 0.50	6.25 ± 0.50	6.25 ± 0.50	7.50 ± 0.58	7.75 ± 0.50
	15	5	34.33 ± 0.40	32.63 ± 0.82	10.20 ± 0.01	11.72 ± 0.01	6.25 ± 0.50	6.50 ± 0.58	6.25 ± 0.50	6.50 ± 0.58	4.50 ± 0.58	4.75 ± 0.50	3.50 ± 0.58	3.75 ± 0.50	4.50 ± 0.58	4.50 ± 0.58	6.25 ± 0.50	6.00 ± 0.01
		10	34.49 ± 0.92	34.90 ± 0.58	11.28 ± 0.14	12.00 ± 0.28	7.00 ± 0.01	7.50 ± 0.58	7.25 ± 0.50	7.50 ± 0.58	4.75 ± 0.50	5.00 ± 0.82	5.25 ± 0.50	5.50 ± 0.58	5.50 ± 0.58	5.75 ± 0.50	7.25 ± 0.50	7.50 ± 0.58
		15	35.54 ± 0.73	36.07 ± 0.57	12.30 ± 0.31	15.41 ± 0.69	8.25 ± 0.50	8.50 ± 0.58	7.50 ± 0.58	7.75 ± 0.50	5.50 ± 0.58	5.75 ± 0.50	6.25 ± 0.50	6.50 ± 0.58	6.25 ± 0.50	6.50 ± 0.58	7.00 ± 0.01	7.25 ± 0.50
Deep Fried (180°C)		10		27.86 ± 1.23		42.35 ± 1.25		5.00 ± 0.82		3.25 ± 0.50		3.75 ± 0.96		2.75 ± 0.96		7.25 ± 0.96		4.50 ± 0.58
C.O with A.F. A. (180°C)		10		22.54 ± 0.78		11.84 ± 0.35		6.25 ± 0.50		6.00 ± 0.82		3.75 ± 0.50		3.25 ± 0.50		4.25 ± 0.50		5.25 ± 0.50
C.O with B.S. A. (180°C)		10		12.91 ± 0.13		10.59 ± 0.24		6.25 ± 0.50		6.75 ± 0.50		2.50 ± 0.58		3.25 ± 0.50		3.50 ± 0.58		5.00 ± 0.01

Potato wedges																		
Temperature (°C)	Air velocity (m/s)	Time (minute)	Hardness (N)		Cutting hardness (N)		Color		Appearance		Crispiness		Internal texture		Oiliness		General sensory appreciation	
			Tray position															
			Bottom	Middle	Bottom	Middle	Bottom	Middle	Bottom	Middle	Bottom	Middle	Bottom	Middle	Bottom	Middle	Bottom	Middle
175	9	5	13.47 ± 1.00	15.07 ± 0.68	7.41 ± 1.15	7.23 ± 1.34	6.25 ± 0.50	6.00 ± 0.82	5.50 ± 0.58	5.75 ± 0.50	1.50 ± 0.58	1.25 ± 0.50	2.25 ± 0.50	2.00 ± 0.01	4.50 ± 0.58	5.50 ± 0.58	3.50 ± 0.58	3.75 ± 0.50
		10	15.21 ± 1.40	16.48 ± 1.04	8.63 ± 0.91	8.26 ± 0.93	6.75 ± 0.50	6.50 ± 0.58	6.50 ± 0.58	6.75 ± 0.50	3.00 ± 0.82	3.00 ± 0.82	2.50 ± 0.58	2.50 ± 0.58	5.00 ± 0.82	5.00 ± 0.82	4.50 ± 0.58	4.00 ± 0.82
		15	16.08 ± 0.68	17.29 ± 0.35	9.65 ± 1.04	9.30 ± 0.69	6.75 ± 0.50	6.50 ± 0.58	6.50 ± 0.58	6.25 ± 0.96	4.25 ± 0.50	4.50 ± 0.58	4.00 ± 0.82	4.00 ± 0.82	5.75 ± 0.50	5.75 ± 0.50	5.50 ± 0.58	5.50 ± 0.58
	12	5	14.21 ± 0.82	16.07 ± 0.53	8.99 ± 1.52	7.76 ± 1.70	5.75 ± 0.50	6.00 ± 0.82	5.75 ± 0.50	5.75 ± 0.50	2.50 ± 0.58	2.75 ± 0.50	2.75 ± 0.50	2.75 ± 0.50	4.00 ± 0.82	4.00 ± 0.82	4.00 ± 0.01	4.75 ± 0.50
		10	15.67 ± 0.41	19.42 ± 1.67	9.13 ± 0.99	9.24 ± 0.84	7.00 ± 0.82	7.25 ± 0.50	7.00 ± 0.82	7.00 ± 0.82	3.25 ± 0.96	3.50 ± 0.58	4.50 ± 0.58	4.50 ± 0.58	5.25 ± 0.50	5.25 ± 0.50	7.00 ± 0.01	6.50 ± 0.58
		15	16.65 ± 1.09	20.36 ± 0.19	10.20 ± 0.80	9.58 ± 1.00	7.25 ± 0.50	7.50 ± 0.58	7.25 ± 0.50	7.25 ± 0.50	5.50 ± 0.58	5.25 ± 0.50	5.75 ± 0.50	5.75 ± 0.50	6.25 ± 0.50	5.75 ± 0.50	7.00 ± 0.01	6.75 ± 0.50
	15	5	14.94 ± 0.64	17.29 ± 0.61	9.09 ± 0.64	8.06 ± 1.09	6.25 ± 0.50	6.50 ± 0.58	7.50 ± 0.58	7.50 ± 0.58	5.00 ± 0.82	4.50 ± 0.58	3.75 ± 0.50	3.75 ± 0.50	4.50 ± 0.58	4.50 ± 0.58	5.00 ± 0.82	7.00 ± 0.01
		10	16.58 ± 0.61	20.25 ± 0.76	9.98 ± 1.38	8.86 ± 1.09	7.25 ± 0.50	7.00 ± 0.01	7.50 ± 0.58	7.50 ± 0.58	5.00 ± 0.82	5.00 ± 0.82	5.50 ± 0.58	5.50 ± 0.58	5.75 ± 0.50	5.50 ± 0.58	7.50 ± 0.58	7.50 ± 0.58
		15	17.27 ± 0.59	23.38 ± 1.10	12.81 ± 1.59	11.90 ± 2.63	7.25 ± 0.50	7.50 ± 0.58	7.50 ± 0.58	7.50 ± 0.58	5.25 ± 0.50	5.50 ± 0.58	6.50 ± 0.58	6.50 ± 0.58	6.50 ± 0.58	5.25 ± 0.96	7.00 ± 0.82	8.50 ± 0.58
200	9	5	15.64 ± 0.85	19.17 ± 0.70	12.72 ± 1.44	9.09 ± 0.82	5.50 ± 0.58	7.50 ± 0.58	5.75 ± 0.50	5.75 ± 0.50	1.50 ± 0.58	1.50 ± 0.58	2.25 ± 0.50	2.25 ± 0.50	4.75 ± 0.50	4.75 ± 0.50	4.25 ± 0.50	9.00 ± 0.01
		10	16.16 ± 0.79	21.72 ± 1.37	13.81 ± 0.95	15.81 ± 1.13	6.75 ± 0.50	7.25 ± 0.50	6.25 ± 0.50	6.25 ± 0.50	2.50 ± 0.58	2.50 ± 0.58	2.00 ± 0.82	2.00 ± 0.82	2.00 ± 0.82	2.00 ± 0.82	4.75 ± 0.50	9.00 ± 0.01
		15	17.45 ± 0.33	23.57 ± 0.45	14.78 ± 0.93	17.59 ± 1.53	7.00 ± 0.82	7.00 ± 0.82	6.75 ± 0.50	6.75 ± 0.50	4.00 ± 0.01	4.00 ± 0.01	3.25 ± 0.50	3.25 ± 0.50	4.00 ± 0.01	4.00 ± 0.01	5.50 ± 0.58	8.75 ± 0.50
	12	5	16.27 ± 0.87	20.80 ± 0.51	13.92 ± 1.25	14.07 ± 1.33	6.25 ± 0.50	7.75 ± 0.50	5.50 ± 0.58	5.50 ± 0.58	4.25 ± 0.50	4.25 ± 0.50	4.00 ± 0.82	4.00 ± 0.82	2.25 ± 0.50	2.25 ± 0.50	4.50 ± 0.58	8.75 ± 0.50
		10	18.00 ± 0.97	23.47 ± 1.30	14.77 ± 0.93	17.57 ± 0.74	7.00 ± 0.01	7.00 ± 0.01	5.75 ± 0.96	5.75 ± 0.96	5.00 ± 0.01	5.00 ± 0.01	4.75 ± 0.50	4.75 ± 0.50	3.00 ± 0.82	3.00 ± 0.82	7.25 ± 0.50	8.50 ± 0.58
		15	19.34 ± 0.46	25.43 ± 0.73	16.78 ± 1.77	17.28 ± 1.45	7.75 ± 0.50	7.75 ± 0.50	7.00 ± 0.82	7.00 ± 0.82	6.00 ± 0.01	6.00 ± 0.01	5.25 ± 0.96	5.25 ± 0.96	4.25 ± 0.50	4.25 ± 0.50	7.75 ± 0.50	8.50 ± 0.58
	15	5	17.82 ± 1.18	22.19 ± 0.29	14.51 ± 0.78	14.07 ± 0.87	8.25 ± 0.50	8.25 ± 0.50	7.25 ± 0.50	7.25 ± 0.50	5.00 ± 0.01	5.00 ± 0.01	4.75 ± 0.96	4.75 ± 0.96	2.25 ± 0.50	2.25 ± 0.50	8.75 ± 0.50	8.00 ± 0.82
		10	19.04 ± 0.69	25.87 ± 0.67	16.29 ± 1.32	17.27 ± 0.97	7.75 ± 0.50	6.75 ± 0.96	6.50 ± 0.58	6.50 ± 0.58	5.75 ± 0.50	5.75 ± 0.50	5.75 ± 0.50	5.75 ± 0.50	4.00 ± 0.82	4.00 ± 0.82	7.75 ± 0.50	6.50 ± 0.58
		15	20.11 ± 0.46	27.27 ± 0.49	17.32 ± 1.44	17.74 ± 0.49	6.75 ± 0.50	6.75 ± 0.50	6.25 ± 0.50	6.25 ± 0.50	5.50 ± 0.58	5.50 ± 0.58	4.25 ± 0.96	4.25 ± 0.96	3.25 ± 0.50	3.25 ± 0.50	7.50 ± 0.58	6.25 ± 0.50
Deep Fried (180°C)		10		13.26 ± 1.11		41.73 ± 1.55		4.25 ± 0.50		3.25 ± 0.50		3.75 ± 0.50		4.00 ± 0.82		6.75 ± 0.50		3.75 ± 0.50
C.O with A.F.A. (180°C)		10		14.58 ± 0.94		13.30 ± 0.45		3.75 ± 0.50		3.25 ± 0.50		2.25 ± 0.50		2.75 ± 0.50		3.50 ± 0.58		3.75 ± 0.50
C.O with B.S.A. (180°C)		10		14.39 ± 1.72		16.29 ± 1.60		4.00 ± 0.01		3.00 ± 0.82		1.50 ± 0.58		3.00 ± 0.82		4.00 ± 0.82		3.50 ± 0.58

Abbreviations: A.F.A., air fry accessory; B.S.A., baking sheet accessory; C.O., conventional oven.

**TABLE 7:** ANOVA results for hardness, cutting hardness, and sensory attributes (color, appearance, crispiness, internal texture, oiliness and general appreciation) of French fries and potato wedges cooked under different processing conditions.

French fries																	
Source	DF	Hardness (N)		Cutting hardness (N)		Color		Appearance		Crispiness		Internal texture		Oiliness		General sensory appreciation	
		Sum of squares	*p*	Sum of squares	*p*	Sum of squares	*p*	Sum of squares	*p*	Sum of squares	*p*	Sum of squares	*p*	Sum of squares	*p*	Sum of squares	*p*
Modal	12	127.20	< 0.0001	35.61	< 0.0001	15.03	0.0005	14.30	< 0.0001	65.57	< 0.0001	64.16	< 0.0001	51.46	< 0.0001	65.63	< 0.0001
A	1	21.32	0.0001	9.68	< 0.0001	4.59	0.0003	1.63	0.0074	36.88	< 0.0001	36.26	< 0.0001	0.0104	0.8996	19.71	< 0.0001
B	1	11.11	0.0027	8.09	< 0.0001	0.0156	0.8053	4.88	< 0.0001	2.64	0.0156	0.0625	0.6479	36.50	< 0.0001	6.25	< 0.0001
C	1	77.62	< 0.0001	11.33	< 0.0001	6.25	< 0.0001	4.82	< 0.0001	19.71	< 0.0001	18.38	< 0.0001	10.01	0.0006	13.13	< 0.0001
D	1	0.6400	0.4271	0.3906	0.2697	0.3906	0.2251	0.1406	0.3970	0.3906	0.3256	0.3403	0.2914	0.2101	0.5724	5.44	< 0.0001
AB	1	0.1700	0.6807	0.6534	0.1571	0.1667	0.4239	0.7526	0.0578	0.2109	0.4679	0.1667	0.4575	0.8438	0.2628	17.09	< 0.0001
AC	1	0.5968	0.4429	1.48	0.0379	1.13	0.0451	0.0156	0.7762	4.00	0.0038	0.5625	0.1783	2.07	0.0856	0.0977	0.3917
AD	1	1.45	0.2359	0.4056	0.2610	0.0938	0.5474	0.0026	0.9075	0.0026	0.9354	0.0104	0.8518	0.0104	0.8996	3.57	< 0.0001
BC	1	6.51	0.0168	0.7668	0.1267	1.38	0.0283	0.4401	0.1404	0.5859	0.2311	5.04	0.0004	0.8438	0.2628	0.0651	0.4832
BD	1	2.57	0.1188	2.09	0.0155	0.0434	0.6816	0.0017	0.9244	0.0434	0.7408	0.1736	0.4484	0.0156	0.8772	0.1736	0.2564
CD	1	2.31	0.1384	0.3725	0.2808	0.1276	0.4834	0.0026	0.9075	0.0026	0.9354	0.0938	0.5763	0.0417	0.8009	0.0234	0.6729
A^2^	1	1.15	0.2907	0.1105	0.5534	0.0035	0.9075	0.0217	0.7376	1.06	0.1111	2.72	0.0056	0.6806	0.3133	0.0425	0.5701
C^2^	1	1.76	0.1930	0.2415	0.3830	0.8342	0.0816	1.61	0.0078	0.0425	0.7433	0.3472	0.2867	0.2222	0.5615	0.0425	0.5701
Residual	23	22.52		7.02		5.78		4.34		8.91		6.71		14.73		2.95	
Cor total	35	149.71		42.64		20.81		18.64		74.48		70.87		66.19		68.58	

Potato wedges																	
Source	DF	Hardness (N)		Cutting hardness (N)		Color		Appearance		Crispiness		Internal texture		Oiliness		General sensory appreciation	
		Sum of squares	*p*	Sum of squares	*p*	Sum of squares	*p*	Sum of squares	*p*	Sum of squares	*p*	Sum of squares	*p*	Sum of squares	*p*	Sum of squares	*p*
Modal	12	422.57	< 0.0001	426.54	< 0.0001	9.15	0.0140	12.43	0.0002	65.62	< 0.0001	58.22	< 0.0001	41.94	< 0.0001	76.83	0.0002
A	1	51.60	< 0.0001	23.25	0.0001	2.19	0.0085	4.38	< 0.0001	35.65	< 0.0001	34.44	< 0.0001	0.6667	0.2395	15.44	0.0012
B	1	114.20	< 0.0001	331.91	< 0.0001	2.01	0.0112	1.89	0.0035	2.01	0.0055	0.1406	0.4974	29.34	< 0.0001	18.78	0.0005
C	1	72.63	< 0.0001	60.20	< 0.0001	1.63	0.0208	2.34	0.0015	20.63	< 0.0001	14.65	< 0.0001	6.77	0.0008	7.32	0.0186
D	1	155.00	< 0.0001	0.0003	0.9860	0.1736	0.4259	0.0017	0.9224	1.421E‐14	1.0000	0.0017	0.9396	0.0278	0.8075	9.51	0.0083
AB	1	0.3675	0.2358	0.4213	0.5372	0.0651	0.6243	1.15	0.0186	0.5859	0.1113	0.0026	0.9260	1.04	0.1448	6.25	0.0283
AC	1	1.72	0.0149	0.0431	0.8431	0.3164	0.2851	1.89	0.0035	4.79	< 0.0001	0.0977	0.5711	1.41	0.0924	1.13	0.3303
AD	1	4.78	0.0002	0.2360	0.6436	0.4401	0.2096	0.0026	0.9051	0.0026	0.9130	0.0026	0.9260	0.8437	0.1875	5.75	0.0347
BC	1	0.1335	0.4705	2.81	0.1195	1.38	0.0320	0.0938	0.4768	0.4401	0.1646	5.75	0.0002	0.5859	0.2693	5.27	0.0423
BD	1	15.52	< 0.0001	3.54	0.0825	0.1111	0.5231	0.0017	0.9224	0.0000	1.0000	0.0017	0.9396	0.2500	0.4671	4.00	0.0740
CD	1	5.67	< 0.0001	2.81	0.1195	0.7526	0.1050	0.0104	0.8116	0.0234	0.7435	0.0026	0.9260	0.3151	0.4150	2.19	0.1793
A^2^	1	0.0342	0.7136	0.5689	0.4741	0.0425	0.6919	0.6328	0.0729	1.46	0.0155	3.02	0.0040	0.6806	0.2348	0.7300	0.4320
C^2^	1	0.9135	0.0674	0.7585	0.4093	0.0425	0.6919	0.0313	0.6801	0.0425	0.6596	0.1050	0.5570	0.0009	0.9656	0.4592	0.5321
Residual	23	5.70		24.70		6.08		4.12		4.91		6.80		10.52		26.25	
Cor total	35	428.28		451.24		15.22		16.55		70.53		65.02		52.45		103.08	

*Note:* A: Air velocity, B: Temperature, C: Time, D: Location (Tray position), Different letters in the same column are significant at *p* < 0.05.

**TABLE 8:** Regression coefficients of coded factors for hardness, cutting hardness, and sensory quality attributes (color, appearance, crispiness, internal texture, oiliness and general appreciation) of French fries and potato wedges.

French fries								
	Coefficient estimate							
	Hardness (N)	Cutting hardness (N)	Color	Appearance	Crispiness	Internal texture	Oiliness	General sensory appreciation
Intercept	33.21	10.61	7.07	6.55	4.24	4.51	3.95	6.53
A	0.9425	0.6350	0.4375	0.2604	1.24	1.23	−0.0208	0.9062
B	0.5556	0.4742	0.0208	0.3681	−0.2708	0.0417	1.01	0.4167
C	1.80	0.6871	0.5104	0.4479	0.9062	0.8750	0.6458	0.7396
D	0.1333	0.1042	0.1042	0.0625	0.1042	0.0972	0.0764	0.3889
AB	0.0842	0.1650	−0.0833	0.1771	−0.0938	−0.0833	0.1875	−0.8437
AC	−0.1931	0.3044	0.2656	0.0313	−0.5000	−0.1875	0.3594	0.0781
AD	−0.2458	0.1300	−0.0625	0.0104	0.0104	−0.0208	−0.0208	−0.3854
BC	−0.5208	0.1788	0.2396	0.1354	0.1563	0.4583	0.1875	−0.0521
BD	−0.2672	0.2408	0.0347	0.0069	0.0347	0.0694	0.0208	−0.0694
CD	−0.3100	−0.1246	0.0729	−0.0104	0.0104	−0.0625	−0.0417	0.0312
A^2^	−0.3783	0.1175	0.0208	0.0521	−0.3646	−0.5833	0.2917	−0.0729
C^2^	0.4692	0.1738	−0.3229	−0.4479	0.0729	−0.2083	0.1667	−0.0729

Potato Wedges								
	Coefficient estimate							
	Hardness (N)	Cutting hardness (N)	Color	Appearance	Crispiness	Internal texture	Oiliness	General sensory appreciation
Intercept	19.03	12.65	7.07	6.42	4.39	4.58	4.12	6.93
A	1.47	0.9842	0.3021	0.4271	1.22	1.20	−0.1667	0.8021
B	1.78	3.04	0.2361	−0.2292	0.2361	−0.0625	−0.9028	0.7222
C	1.74	1.58	0.2604	0.3125	0.9271	0.7812	0.5312	0.5521
D	2.07	−0.0031	0.0694	0.0069	0.0000	−0.0069	0.0278	0.5139
AB	0.1237	0.1325	0.0521	−0.2188	0.1562	0.0104	−0.2083	−0.5104
AC	0.3275	−0.0519	−0.1406	−0.3437	−0.5469	−0.0781	0.2969	−0.2656
AD	0.4463	−0.0992	−0.1354	−0.0104	−0.0104	0.0104	−0.1875	−0.4896
BC	0.0746	0.3421	−0.2396	−0.0625	−0.1354	−0.4896	−0.1563	−0.4688
BD	0.6567	0.3136	0.0556	−0.0069	0.0000	0.0069	0.0833	0.3333
CD	0.4863	0.3421	−0.1771	−0.0208	0.0313	0.0104	−0.1146	−0.3021
A^2^	−0.0654	−0.2667	−0.0729	0.2813	−0.4271	−0.6146	0.2917	−0.3021
C^2^	−0.3379	−0.3079	−0.0729	−0.0625	0.0729	−0.1146	0.0104	−0.2396

*Note:* A: Air velocity, B: Temperature, C: Time, D: Location (Tray position).

As shown in Table 6, hardness and cutting hardness values increased progressively with increasing processing intensity, particularly under higher temperatures, longer processing times, and greater air velocities. In French fries, hardness increased from approximately 27–31 N under mild processing conditions to values exceeding 35 N under more intensive conditions, while cutting hardness exhibited a similar increasing trend. A comparable pattern was observed for potato wedges, in which hardness values increased from approximately 13–16 N at lower processing intensities to more than 25 N under severe processing conditions.

From a sensory perspective, crispiness and appearance scores improved with increasing process severity up to an optimum range. In French fries, crispiness scores increased from approximately 4.75–6.00 under mild conditions to 7.50–8.50 under higher‐intensity treatments. A similar tendency was observed in potato wedges, where crispiness scores exceeded 7.5 under optimized conditions. In contrast, oiliness scores consistently decreased under air jet impingement conditions compared with deep‐fat fried samples, indicating reduced oil absorption. Overall sensory acceptability followed a similar trend, with the highest scores generally obtained under intermediate‐to‐high processing conditions.

The ANOVA results presented in Table 7 confirmed that air velocity (A), temperature (B), and processing time (C) significantly affected both hardness and cutting hardness in French fries and potato wedges (*p* < 0.05). Among these variables, temperature and processing time exerted the strongest effects, indicating that thermal load and exposure duration were the primary factors governing structural development.

For potato wedges, tray position (D) also significantly influenced hardness, suggesting that spatial heat distribution within the oven affected product structure (*p* < 0.05). However, its effect on cutting hardness was not statistically significant (*p* > 0.05), indicating that mechanical resistance was less sensitive to vertical positioning within the oven cavity.

Whereas ANOVA identified the statistical significance of the processing variables, the regression coefficients presented in Table 8 provided additional insight into the magnitude and direction of these effects. Processing time (C) exhibited the strongest positive effect on hardness development, particularly in French fries, whereas temperature (B) exerted a more pronounced influence on cutting hardness in potato wedges.

Crispiness was positively associated with air velocity and processing time, confirming that intensified convective heat transfer promoted crust formation. In contrast, oiliness exhibited negative or negligible relationships with the processing variables, supporting the observed reduction in oil absorption under air jet impingement conditions. Overall sensory acceptability was mainly influenced by processing time and air velocity, indicating that sufficient exposure to high‐velocity hot air is critical for achieving desirable sensory quality. These findings are consistent with previous studies. Teruel et al. (2015) reported distinct differences in crust formation and internal texture development between deep‐fat fried and air‐fried potato products, noting more rapid initial softening during deep frying but greater internal hardness in air‐fried samples due to reduced starch gelatinization and delayed heat penetration. Similarly, Arafat (2014) and Tian et al. (2017) demonstrated that air‐fried potato products can achieve sensory acceptance comparable to conventional French fries in terms of crispiness, appearance, taste, and aroma, while also providing advantages such as reduced oil absorption, improved color development, and more controlled hardness evolution.

Air jet impingement technology provides a highly efficient heat transfer environment that enhances crust formation and crispiness development through intensified convective conditions while simultaneously limiting oil absorption compared with conventional deep‐fat frying. Consequently, this technology offers a favorable balance between structural integrity and sensory quality, supporting its potential as an effective alternative to traditional frying systems for the production of reduced‐fat potato products with improved quality characteristics.

**FIGURE 3:**
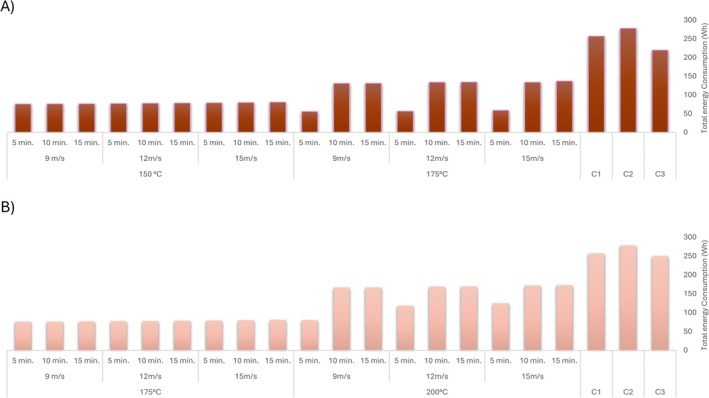
Total energy consumption of (A) French Fries, (B) potato wedges, and control samples cooked under different processing conditions. C1: Conventional oven with air fry accessory (180°C), C2: Conventional oven with baking tray (180°C), C3: Deep frying (180°C).

The energy consumption of the air jet impingement oven equipped with the air‐fry function was compared with that of a conventional oven and deep‐fat frying (Figure 3). Power consumption increased with increasing oven temperature and air velocity under impingement‐assisted air frying conditions. Nevertheless, the total energy consumption required to achieve comparable product quality was lower for the air jet impingement oven than for both deep‐fat frying and conventional oven cooking. This reduction can largely be attributed to the high heat transfer efficiency of the impingement jets, which enabled shorter processing times and improved energy utilization efficiency.

### 3.4 Determination of Optimum Cooking/Frying Parameters and Acrylamide Content in Potatoes

Overall, the quadratic regression models developed for both French Fries and potato wedges exhibited good adequacy, as indicated by the close agreement between *R* ^2^ and *R* ^2^_adj_ values (Table 9). In addition, all Adequate Precision values exceeded the recommended threshold of 4, confirming sufficient signal‐to‐noise ratios and reliable predictive capability within the experimental domain.

**TABLE 9:** Summary of fit statistics for quadratic regression models for french fries and potato wedges.

French fries																
	Moisture content (%)	Cooking loss (%)	Crust thickness (mm)	b*	BI	Oil content (%, DM)	Oil absorption (%, DM)	TBARS (mg malonaldehyde/kg product)	Hardness (N)	Cutting hardness (N)	Color	Appearance	Crispiness	Internal texture	Oiliness	General sensory appreciation
*R* ^2^	0.9281	0.9683	0.8973	0.6826	0.7722	0.9602	0.9604	0.8989	0.8496	0.8353	7.30	0.7671	0.8804	0.9053	0.7775	0.9570
*R* ^2^_adj_	0.8906	0.9517	0.8437	0.5170	0.6534	0.9394	0.9397	0.8461	0.7711	0.7493	0.5772	0.6456	0.8180	0.8559	0.6614	0.9346
C.V. %	5.82	7.98	9.90	12.28	13.96	9.33	14.54	7.33	2.97	5.11	7.30	6.91	15.37	13.55	18.80	5.57
Adeq precision	19.5844	28.1023	16.3209	7.7896	9.8652	26.5988	26.6451	17.6262	13.8570	12.5858	8.2510	8.7638	13.4825	16.3011	9.3795	27.8913

Potato wedges																
	Moisture content (%)	Cooking loss (%)	Crust thickness (mm)	b*	BI	Oil content (%, DM)	Oil absorption (%, DM)	TBARS (mg malonaldehyde/kg product)	Hardness (N)	Cutting hardness (N)	Color	Appearance	Crispiness	Internal texture	Oiliness	General sensory appreciation
*R* ^2^	0.9281	0.9558	0.9696	0.9396	0.9274	0.9378	0.9192	0.9116	0.9867	0.9453	0.6008	0.7509	0.9303	0.8954	0.7995	0.7453
*R* ^2^_adj_	0.8906	0.9328	0.9537	0.9080	0.8895	0.9054	0.8771	0.8655	0.9797	0.9167	0.3926	0.6210	0.8940	0.8408	0.6949	0.6125
C.V. %	4.13	9.91	6.74	4.11	8.09	10.20	20.72	6.25	2.65	8.45	7.37	6.45	11.13	13.30	15.65	16.26
Adeq precision	20.6464	22.2628	27.4064	21.5532	19.1956	21.6683	19.2889	18.4622	47.1979	19.7869	7.5434	8.3399	17.3031	15.1730	10.5196	9.9763

For the optimization stage, the selected response variables for French Fries included TBARS value as an indicator of lipid oxidation, oil absorption (%) as an undesirable quality attribute, cutting hardness, and yellowness (b), which are directly related to consumer acceptability. For potato wedges, crust thickness was additionally included as a response variable due to its contribution to sensory preference.

**FIGURE 4:**
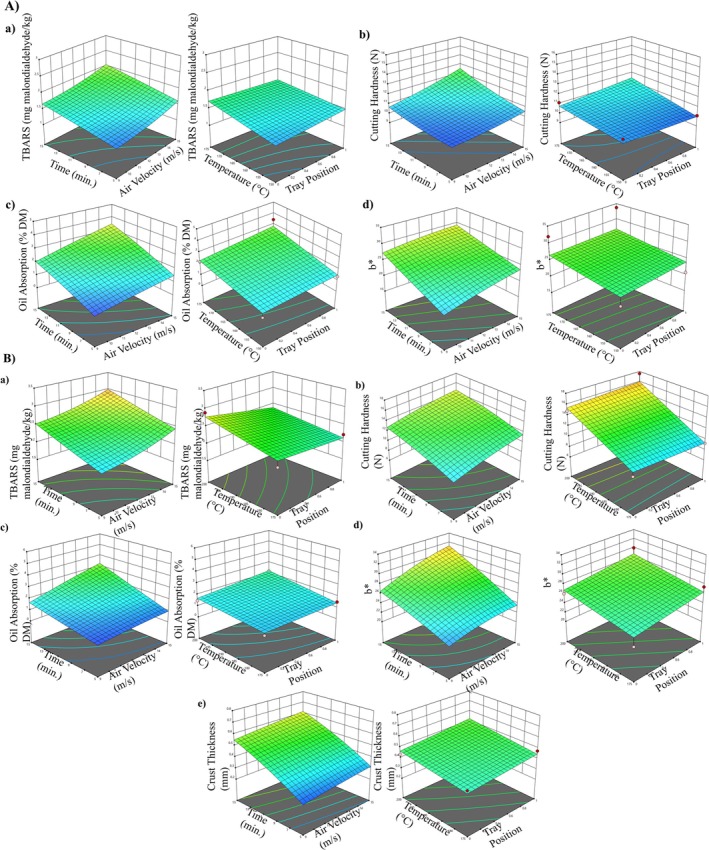
Response surface plots showing the effects of process parameters on the response variables of (A) French Fries and (B) Potato Wedges; (a) TBARS value (mg malonaldehyde/kg), (b) Cutting Hardness (N), (c) Oil Absorption (% DM), (d) *b* value and (e) Crust Thickness (mm).

The response surface plots presented in Figure 4 clearly demonstrated the interactive effects of air velocity, temperature, cooking/frying time, and tray position on the investigated quality parameters of French Fries (Figure 4A) and potato wedges (Figure 4B). These plots provided a comprehensive visualization of the relationships between processing variables and response behaviors within the experimental domain.

As illustrated in Figure 4A, TBARS values of French Fries increased markedly with increasing air velocity and cooking/frying time. In contrast, the effect of cooking/frying temperature and tray position on TBARS remained relatively limited. The primary determinants for cutting hardness were air velocity and cooking/frying time. Cutting hardness significantly increased with both longer cooking/frying times and higher air velocity. The influence of temperature and tray position on cutting hardness was less pronounced. Although the amount of oil absorption varied primarily with air velocity and cooking/frying time, it generally remained at low levels. It was observed that the uptake showed an increasing trend at the highest air velocity and exhibited slight variations depending on the tray position. The yellowness (b) value, a color parameter that strongly influences consumer preference, showed an increasing trend with higher air velocity and longer cooking/frying time, indicating the formation of a desirable darker and more yellow product.

Similarly, Figure 4B demonstrated that TBARS values of potato wedges increased significantly with increasing air velocity, consistent with the French Fries results. However, the effect of tray position became more pronounced in this product. In terms of cutting hardness, and unlike the French Fries, all process parameters (air velocity, time, temperature, and position) were identified as effective determinants for potato wedges. Specifically, samples cooked for longer durations and at higher temperatures exhibited higher cutting hardness values. Oil absorption values increased with both air velocity and cooking/frying time, while the effects of tray position and temperature remained limited. For the color parameter yellowness (b) value, an increasing trend was observed with higher temperature and longer cooking time, and the effect of air velocity was found to be more noticeable compared to French Fries. Finally, crust thickness, a critical quality parameter additionally evaluated for potato wedges, significantly increased with air velocity and time; the effects of temperature and tray position remained relatively limited. Based on the response surface trends shown in Figure 4, optimization was carried out to determine the most favorable cooking/frying conditions.

During multi‐response optimization, TBARS value, oil absorption, and cutting hardness were set to be minimized within an acceptable range to avoid undercooked or excessively oxidized and hard products. In contrast, the *b* value was maximized to enhance color acceptability. For potato wedges, crust thickness was also maintained within a defined range to balance insufficient and excessive crust formation. Optimization was performed using the desirability function approach in Design‐Expert software.

The desirability‐based optimization yielded high desirability values for both products, indicating reliable optimum solutions. The desirability values were 0.932 for French Fries and 0.822 for potato wedges (Table 10). Based on these results, the optimum cooking conditions were determined as 160°C, 15 m/s air velocity, 15 min, middle tray position for French Fries, and 200°C, 15 m/s air velocity, 5 min, middle tray position for potato wedges.

**TABLE 10:** Optimum cooking/frying conditions determined by considering the response variable analysis results for French Fries and potato wedges.

French fries									
No	Temperature (°C)	Air velocity (m/s)	Time (minute)	Location (tray position)	TBARS (mg malonaldehyde/kg product)	Cutting hardness (N)	Oil absorption (%, DM)	b*	Desirability
1	160.00	15.00	15.00	Middle	1.812	11.529	4.650	29.265	0.932
2	162.17	15.00	15.00	Middle	1.812	11.529	4.632	29.265	0.931
3	164.00	15.00	15.00	Middle	1.812	11.529	4.615	29.265	0.931

Potato Wedges										
No	Temperature (°C)	Air velocity (m/s)	Time (minute)	Location (tray position)	TBARS (mg malonaldehyde/kg product)	Cutting hardness (N)	Oil absorption (%, DM)	b*	Crust thickness (mm)	Desirability
1	200.00	15	5.00	Middle	2.805	26.125	2.862	31.264	3.184	0.822
2	199.97	15	5.11	Middle	2.804	26.126	2.860	31.256	3.185	0.821
3	200.00	15	5.07	Middle	2.810	25.975	2.858	31.237	3.172	0.820

Table 10 presents the results of response variable results for French Fries and potato wedges processed under the optimum cooking/frying conditions determined using the desirability function approach. Samples processed in the air jet impingement oven with air‐fry function consistently exhibited lower oil absorption and TBARS values, while maintaining acceptable textural and sensory properties compared with conventional oven cooking. These improvements may be attributed to the high heat transfer efficiency of the impingement jets, which enabled effective cooking at lower temperatures and shorter processing times.

**FIGURE 5:**
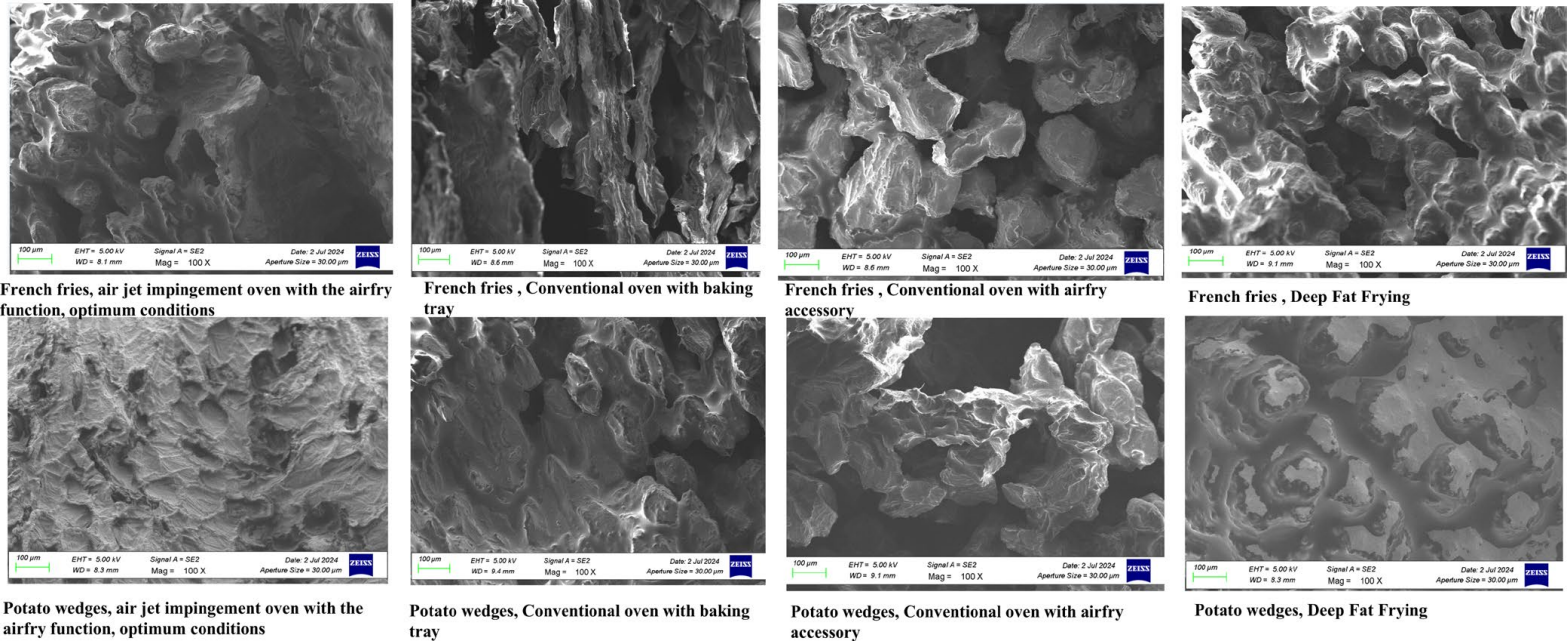
SEM images (100× magnification) of potatoes cooked in deep frying, conventional oven with baking tray, conventional oven with air‐fry accessory, and air jet impingement oven with air‐fry function under optimum cooking/frying conditions.

The SEM images of French Fries and potato wedges cooked under optimum conditions in the air jet impingement oven, conventional oven, and deep fryer are shown in Figure 5. Examination of these images revealed surface flashes in deep‐fried samples due to high oil content, with smaller pore structures compared to other methods. These observations are consistent with texture analysis results (Table 6). In conventional oven cooking, the pore and fibrous structures were more developed when using the air‐fry accessory compared to the oven tray. Notably, potatoes cooked in the air jet impingement oven exhibited a porous and fibrous microstructure without visible lipid deposition, indicating effective frying with minimal oil absorption and supporting the observed reductions in oil content and increased hardness values.

Table 11 presents acrylamide content in French Fries and potato wedges cooked under optimum conditions in the air jet impingement oven and by deep frying. Acrylamide is a chemical formed in carbohydrate‐rich foods subjected to high‐temperature cooking, particularly under low‐humidity conditions. Acrylamide is a heat‐induced Maillard reaction product formed primarily from the reaction between free asparagine and reducing sugars in carbohydrate‐rich foods subjected to high‐temperature, low‐moisture cooking conditions. Potatoes and cereals are primary sources, and acrylamide has been classified by the International Agency for Research on Cancer (WHO 1994) as a Group 2A substance, meaning it is “possibly carcinogenic to humans.” Toxicological studies have demonstrated that acrylamide is neurotoxic, can induce anemia, and exhibits carcinogenic effects in laboratory animals (Backe et al. 2014; Kısabay et al. 2004).

**TABLE 11:** Acrylamide content of potatoes cooked in the air jet impingement oven with air‐fry function and by deep frying.

	French fries		Potato wedges	
	Air jet impingement oven (160°C, 15 m/s, 15 min middle tray position)	Deep frying (180°C, 10 min)	Air jet impingement oven (200°C, 15 m/s, 5 min middle tray position)	Deep frying (180°C, 10 min)
Acrylamide content (mg/kg)	0.280^ab^ ±0.051	0.360^b^ ± 0.066	Not detected^a^	0.670^c^ ± 0.123

*Note:* Different superscript letters indicate significant differences at *p* < 0.05.

In the present study, acrylamide formation was lower in samples processed using the air jet impingement oven than in deep‐fat fried samples (Table 11). In French fries, acrylamide content decreased from 0.360 mg/kg in deep‐fat fried samples to 0.280 mg/kg under optimized air jet impingement conditions, although both treatments were assigned to similar statistical letter groupings. In contrast, potato wedges processed under optimized air jet impingement conditions (200°C, 15 m/s, 5 min, middle tray position) exhibited no detectable acrylamide formation, whereas deep‐fat fried samples showed the highest acrylamide level (0.670 mg/kg) and belonged to a different statistical grouping, indicating a clear difference between treatments.

These findings suggest that optimized impingement processing conditions can substantially suppress acrylamide formation, particularly in potato wedges. The reduced acrylamide generation observed under air jet impingement conditions may be attributed to rapid convective heat transfer and shorter exposure of reducing sugars and free asparagine to high‐temperature, low‐moisture environments, thereby limiting Maillard reaction–driven acrylamide formation. Similar trends were reported by Elitaş et al. (2018), who observed acrylamide levels ranging from 0.33 to 3.7 mg/kg in potato products processed using different frying methods. The present results demonstrate that air jet impingement technology can improve the chemical safety profile of fried potato products while maintaining desirable quality characteristics.

## 4 Conclusion

This study demonstrated that air jet impingement–assisted air frying significantly influences the physicochemical quality and chemical safety of French Fries and potato wedges. Compared with conventional deep frying and convection oven cooking, the air jet impingement oven effectively reduced oil absorption and lipid oxidation while maintaining desirable textural, color, and sensory attributes. The observed reductions in oil absorption and TBARS values were primarily attributed to enhanced heat and mass transfer conditions, which limited oil exposure and oxidative degradation during cooking.

Processing parameters, particularly air velocity, temperature, and cooking time, played a critical role in determining product quality, including moisture retention, crust formation, and color development, as well as overall sensory acceptability. Importantly, acrylamide formation was substantially suppressed under optimized air jet impingement conditions, with levels in potato wedges below the limit of quantification, highlighting the potential of this technology to mitigate harmful Maillard reaction products.

Air jet impingement–assisted air frying offers a promising processing strategy for producing potato products with acceptable quality attributes while effectively reducing oil absorption, lipid oxidation, and acrylamide formation, and improving process efficiency through shorter cooking times and enhanced heat transfer, supporting its potential application in both industrial‐scale production and domestic food processing systems.

## Author Contributions

**Gönül Çavuşoğlu‐Kaplan:** conceptualization, investigation, validation, writing – review and editing. **Özge Filiz:** conceptualization, methodology, validation, writing – original draft, writing – review and editing. **Ahmet Tarık Can:** conceptualization, formal analysis, investigation, methodology, validation, writing – original draft, writing – review and editing. **Selin Demirkan:** conceptualization, formal analysis, investigation, methodology, validation, writing – original draft, writing – review and editing. **Özgül Altay:** conceptualization, methodology, validation, writing – original draft, writing – review and editing. **Figen Kaymak‐Ertekin:** funding acquisition, project administration, supervision, writing – original draft, writing – review and editing.

## Funding

This study was supported by the Scientific and Technological Research Council of Türkiye (TÜBİTAK‐2209B, Project number: 1139B412300178) and Arçelik A.Ş.

## Conflicts of Interest

The authors declare no conflicts of interest.

## Data Availability

The data that support the findings of this study are available from the corresponding author upon reasonable request.
